# Regulating Local Coordination Sphere of Ir Single Atoms at the Atomic Interface for Efficient Oxygen Evolution Reaction

**DOI:** 10.1021/jacs.4c08847

**Published:** 2024-10-08

**Authors:** Ashwani Kumar, Marcos Gil-Sepulcre, Jean Pascal Fandré, Olaf Rüdiger, Min Gyu Kim, Serena DeBeer, Harun Tüysüz

**Affiliations:** † Max Planck Institut für Kohlenforschung, 45470 Mülheim an der Ruhr, Germany; ‡ Max Planck Institute for Chemical Energy Conversion, Stiftstrasse 34-36, D-45470 Mülheim an der Ruhr, Germany; § Beamline Research Division, Pohang Accelerator Laboratory (PAL), Pohang 790-784, South Korea; ∥ IMDEA Materials Institute, Calle Eric Kandel 2, Getafe, Madrid 28906, Spain

## Abstract

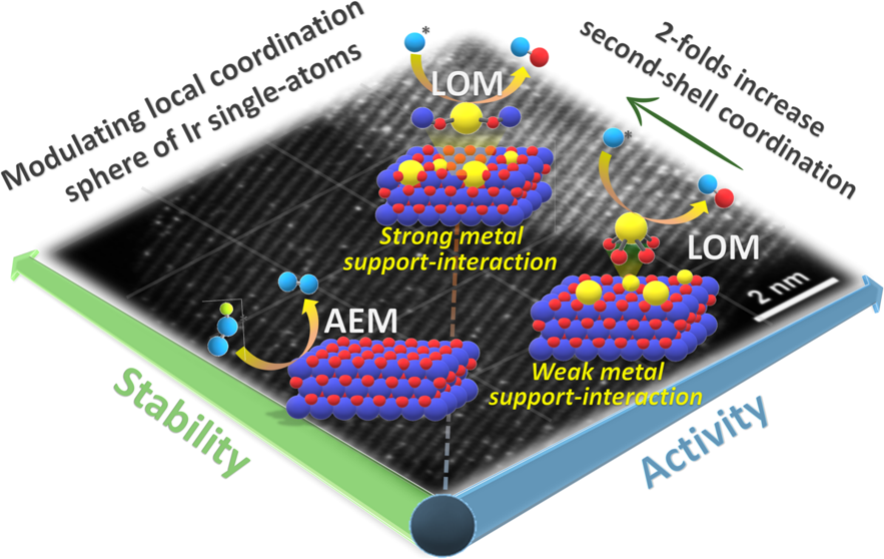

Single-atom catalysts dispersed on an oxide support are essential for overcoming the sluggishness of the oxygen evolution reaction (OER). However, the durability of most metal single-atoms is compromised under harsh OER conditions due to their low coordination (weak metal–support interactions) and excessive disruption of metal-O_lattice_ bonds to enable lattice oxygen participation, leading to metal dissolution and hindering their practical applicability. Herein, we systematically regulate the local coordination of Ir_single-atoms_ at the atomic level to enhance the performance of the OER by precisely modulating their steric localization on the NiO surface. Compared to conventional Ir_single-atoms_ adsorbed on NiO surface, the atomic Ir atoms partially embedded within the NiO surface (Ir_emb_-NiO) exhibit a 2-fold increase in Ir–Ni second-shell interaction revealed by X-ray absorption spectroscopy (XAS), suggesting stronger metal–support interactions. Remarkably, Ir_emb_-NiO with tailored coordination sphere exhibits excellent alkaline OER mass activity and long-term durability (degradation rate: ∼1 mV/h), outperforming commercial IrO_2_ (∼26 mV/h) and conventional Ir_single-atoms_ on NiO (∼7 mV/h). Comprehensive *operando* X-ray absorption and Raman spectroscopies, along with pH-dependence activity tests, identified high-valence atomic Ir sites embedded on the NiOOH surface during the OER followed the lattice oxygen mechanism, thereby circumventing the traditional linear scaling relationships. Moreover, the enhanced Ir–Ni second-shell interaction in Ir_emb_-NiO plays a crucial role in imparting structural rigidity to Ir single-atoms, thereby mitigating Ir-dissolution and ensuring superior OER kinetics alongside sustained durability.

## 1 Introduction

Electrocatalytic water oxidation reaction, denoted as oxygen evolution reaction (OER), plays a pivotal role in various clean energy technologies, including water electrolysis, CO_2_ reduction, and rechargeable metal-air batteries.^1−4^ Unfortunately, the overall power conversion efficiency faces substantial hindrance owing to the sluggish kinetics of the OER, even for the current state-of-the-art OER catalysts (IrO_2_ and RuO_2_).^5^ In addition, the high cost, limited abundance of precious metals, suboptimal mass activity, and inadequate durability further impede their large-scale applications.^6^ Consequently, the exploration of low-noble-metal-containing OER electrocatalysts with high efficiency and sustained stability remains a substantial scientific challenge.

To address these issues, the development of single-atom catalysts (SACs) emerges as one of the most promising strategies for maximizing active metal atom utilization and enhancing metal–support interactions.^7,8^ SACs typically feature atomically dispersed active metal atoms stabilized on the surface of a support, analogous to metalloenzymes in nature.^9^ The catalytic properties of SACs are synergistically determined by the metal single atom and its coordinating atom in the first/second coordination spheres, which strongly regulates the electronic structure of SACs through short/long-range electron delocalization (Figure S1).^10,11^ Currently, the employment of single-site Ir adsorbed on the surface of a transition metal-oxide support via Ir–O_support_ coordination (first coordination sphere) has exhibited unprecedented OER mass activity because of maximum utilization of Ir sites and enhanced Ir–O_support_ bond covalency.^12,13^ Such an intensified covalent nature of the Ir–O_support_ bond in SACs triggers the participation of lattice oxygen during the OER via the lattice oxygen mechanism (LOM), circumventing the formation of *OOH intermediate (traditional adsorbate evolution mechanism: AEM), thus leading to higher activities.^14^ Nonetheless, the LOM pathway is often accompanied by structural destabilization owing to the rupture of atomic Ir–O_lattice_ bonds, depletion of lattice oxygen, and generation of oxygen vacancies, collectively accelerating the dissolution of surface atomic Ir atoms and leading to SACs degradation.^15^

A typical strategy to regulate a single metal–support interaction involves altering the structure and composition of the supports. However, such adjustments may inadvertently affect catalytic activities, complicating the comprehensive understanding of the underlying factors contributing to improved performance.^16^ Moreover, SACs obtained through various synthesis routes often tend to migrate and aggregate during preparation or application, compromising their stability during prolonged catalytic operations.^17^ Hence, enhancing the fundamental stability of single atoms (specifically, severe metal leaching) is intrinsically linked to the overall OER performance of SACs, typically associated with their local coordination environment.^18,19^ Therefore, it is highly desirable to devise a strategy to strengthen the single metal–support interactions via modulating the local coordination environment of active single metals by simply adjusting their steric locations on the support surface without altering the supports. However, achieving precise regulation of the microenvironment of single atoms to optimize the OER performance from both rational structure design and durability perspectives remains conceptually elusive and challenging.

Herein, we present a straightforward and innovative strategy to regulate the local coordination spheres of Ir single atoms at the atomic interface by precisely tuning their steric locations on the NiO surface, revealing substantial impacts on the alkaline OER mass activity and stability. The Ir single atoms adsorbed on the NiO surface (Ir_ads_-NiO) obtained via a conventional synthesis method (electrostatic Ir-ion adsorption on the NiO surface followed by annealing) displayed reduced Ir–Ni coordination in the second coordination sphere. Conversely, atomic Ir sites partially embedded within the NiO surface (Ir_emb_-NiO), synthesized via Ir-ion exchange with Ni-layered double hydroxide (Ni-LDH) surface followed by phase transformation to NiO, and exhibited a 2-fold increase in Ir–Ni second coordination sphere, highlighting strengthened metal–support interactions. Compared to Ir_ads_-NiO, Ir_emb_-NiO with a tailored second coordination sphere exhibited excellent alkaline OER kinetics, achieving high mass activity and sustained durability over 110 h, surpassing commercial IrO_2_ and conventional Ir_ads_-NiO. *Operando* X-ray absorption and Raman spectroscopy, pH-dependence activity tests, and molecular probe analysis collectively revealed that high-valence atomic Ir sites embedded within the NiOOH surface under operating conditions followed the LOM pathway for the OER, overcoming the scaling relationship of the AEM. Furthermore, the higher Ir–Ni second-shell interactions in Ir_emb_-NiO conferred additional structural stability, mitigating Ir-dissolution and enhancing long-term durability of Ir single-atoms under harsh OER conditions.

## 2 Results and Discussion

### 2.1 Synthesis and Structural Characterization

**Figure 1:**
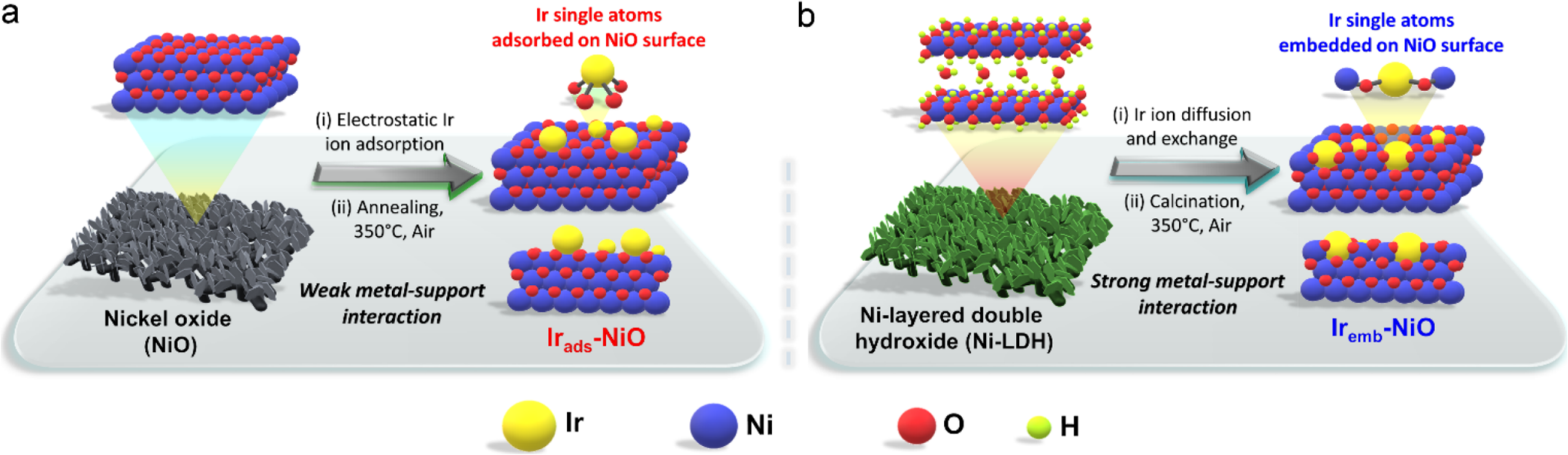
Schematic illustrating the anchoring of Ir single atoms with different steric locations (modulated local coordination spheres) on the NiO surface. (a) Synthesis strategy for obtaining Ir single atoms adsorbed on the NiO surface (Ir_ads_-NiO). (b) Synthesis strategy for stabilizing Ir single atoms partially embedded on the NiO surface (Ir_emb_-NiO).

**Figure 2:**
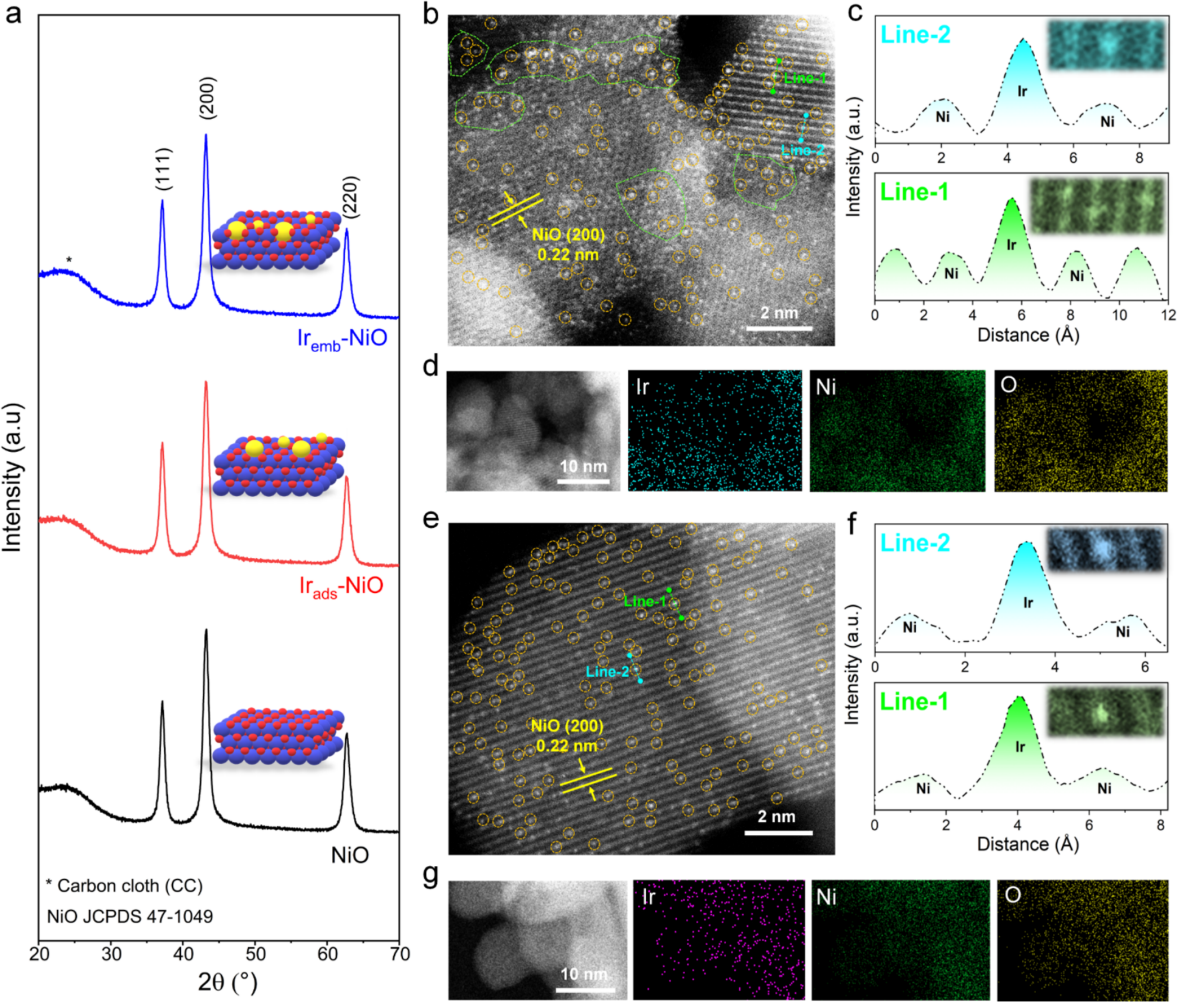
Structural analysis and electron microscopy. (a) XRD patterns of NiO, Ir_ads_-NiO, and Ir_emb_-NiO. (b) Magnified aberration-corrected high-angle annular dark-field scanning transmission electron microscopy (HAADF STEM) image of Ir_ads_-NiO with orange circles showing atomically dispersed Ir atoms and (c) line-scanning intensity profile obtained along lines 1 and 2 in (b). (d) HAADF-STEM image and corresponding EDS map of Ir_ads_-NiO. (e) Magnified aberration-corrected HAADF-STEM image of Ir_emb_-NiO with orange circles indicating singly dispersed Ir atoms and (f) line-scanning intensity profile obtained along lines 1 and 2 in (e). (g) HAADF-STEM image and corresponding EDS maps of Ir_emb_-NiO.

The synthesis strategy for modulating the local coordination spheres of Ir single-atoms by precisely adjusting their steric locations on the NiO surface is schematically illustrated in Figure 1. To synthesize conventional Ir single atoms adsorbed on NiO surface, the NiO support was first prepared by electrodepositing Ni-layered double hydroxide (Ni-LDH) onto a carbon cloth substrate, followed by calcination at 350 °C for 3 h to yield NiO with pure face-centered cubic (FCC) phase (JCPDS: 47-1049), confirmed by the X-ray diffraction (XRD) pattern (Figures 1a and 2a).^20^ Then, the NiO support was immersed in an ethanolic Ir-ion solution (6 mg/mL) to facilitate the electrostatic adsorption of Ir-ions onto the NiO surface, followed by drying and annealing at 350 °C for 2 h to form isolated Ir single atoms adsorbed on the NiO surface (Ir_ads_-NiO).^8^ On the other hand, the atomic Ir atoms partially embedded within the NiO surface (Ir_emb_-NiO) with modulated microenvironment were obtained by immersing the Ni-LDH support directly into an Ir-ion solution (6 mg/mL) for allowing Ir-ion diffusion and exchange with the Ni-LDH surface, followed by calcination and phase transformation at 350 °C for 3 h as illustrated in Figure 1b.

All diffraction peaks in the XRD patterns of both Ir_ads_-NiO and Ir_emb_-NiO were attributed to the NiO phase, suggesting the absence of any Ir-based metals/metal-oxides, while their field-emission scanning electron microscopy (FE-SEM) images depicted similar morphologies with highly rough surfaces (Figures 2a and S2). Energy-dispersive X-ray spectroscopy (EDS) and inductively coupled plasma optical emission spectrometry (ICP-OES) analyses confirmed high Ir loadings of 6.5 and 6.2 wt % for Ir_ads_-NiO and Ir_emb_-NiO, respectively (Figure S3 and Table S1). Aberration-corrected high-angle annular dark-field scanning transmission electron microscopy (HAADF-STEM) images, featuring sub-Å resolution, depicted isolated bright spots of Ir atoms due to *Z*-contrast relative to the support in both Ir_ads_-NiO and Ir_emb_-NiO samples, thereby validating the atomic dispersion of Ir atoms on the surface of NiO for both Ir-containing samples (Figures S4 and 2b,e).^20^ In addition, the atomic Ir atoms in Ir_emb_-NiO are well-aligned with the lattice of NiO, indicative of a pronounced degree of orderliness, contrasting with Ir_ads_-NiO where a significant fraction of Ir single-atoms exhibit considerable disorder. Moreover, the existence of atomic Ir atoms across the NiO support in both samples was corroborated by the intensity profiles and corresponding EDS elemental mappings (Figure 2c,d,f,g).

### 2.2 Local Coordination and Electronic Structure Recognition

**Figure 3:**
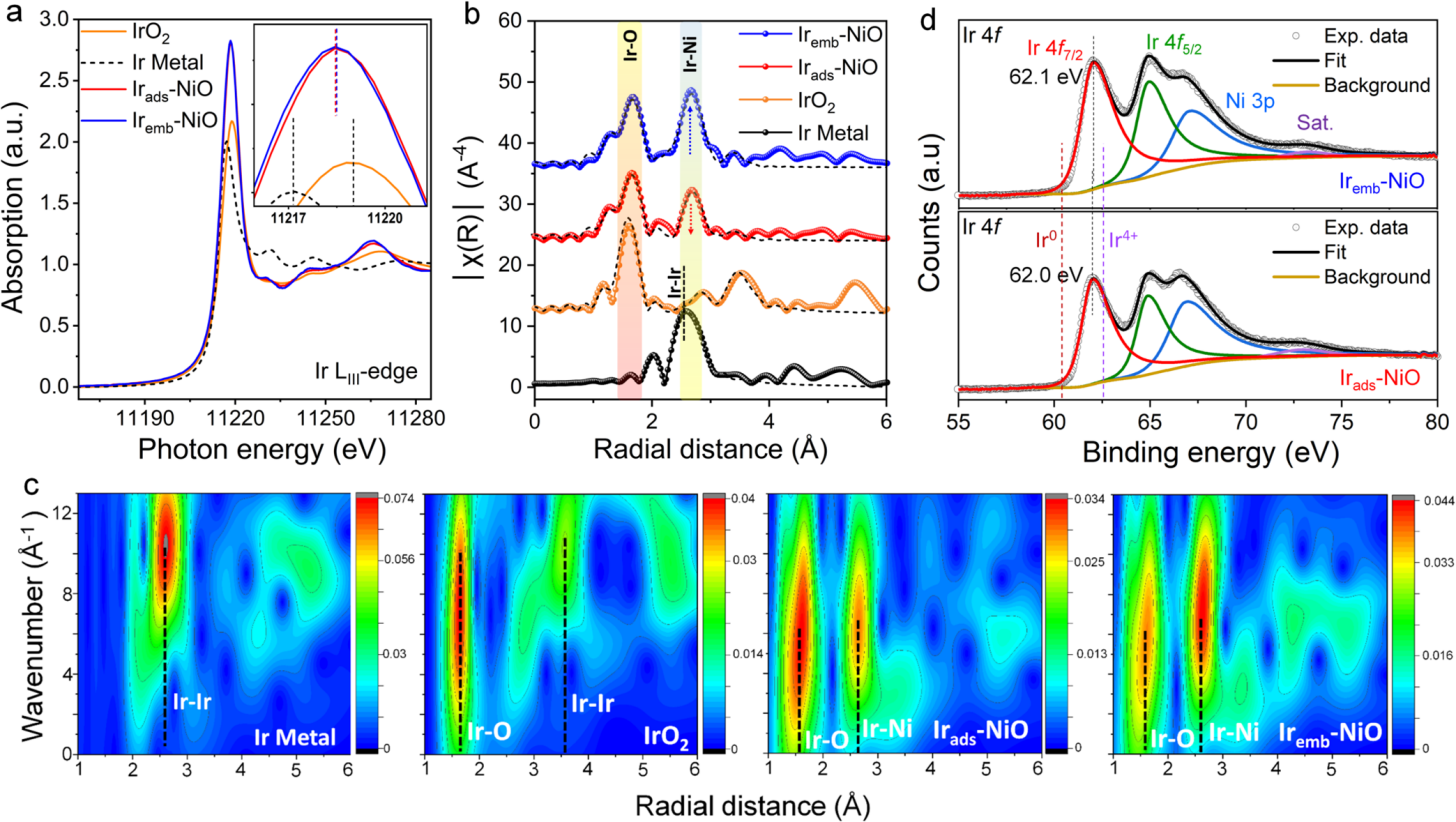
Electronic structure analysis of Ir_ads_-NiO and Ir_emb_-NiO. (a) Experimental Ir-*L*_3_ edge XANES spectra of Ir_ads_-NiO and Ir_emb_-NiO with other reference samples. The inset zooms in over the white line feature. (b) Experimental Ir-*L*_3_ edge FT-EXAFS spectra of Ir_ads_-NiO and Ir_emb_-NiO with other reference samples. (c) Wavelet transform EXAFS (WT-EXAFS) of Ir_ads_-NiO and Ir_emb_-NiO along with reference samples at Ir-*L*_3_ edge. (d) Fitted deconvoluted high-resolution Ir 4f XPS spectra of Ir_ads_-NiO and Ir_emb_-NiO.

The detailed electronic structure and local coordination environments of Ir_ads_-NiO and Ir_emb_-NiO were further investigated by X-ray absorption spectroscopy (XAS) and X-ray photoelectron spectroscopy (XPS) measurements.^21^ The Ni *K*-edge X-ray absorption near-edge structures (XANES) of Ir_emb_-NiO and Ir_ads_-NiO are consistent with the pristine NiO, where Ni sites display an *Oh* geometry and oxidation state II (Figure S5a). This was also confirmed by Ni *K*-edge Fourier-transformed *k*^3^-weighted extended X-ray absorption fine structure analysis (FT-EXAFS), which provides additional information about the coordination environment of the absorbing metal center.^22^ The Ni *K*-edge FT-EXAFS spectra shown in Figure S5b were dominated by contribution from Ni–O and Ni–Ni scattering vectors at 2.07 and 2.95 Å, respectively, attributed to the first and second shells of NiO (Figure S6a and Table S2). The negligible changes with respect to the bare NiO corroborate that the surface Ir-functionalization barely influences the bulk NiO substrate, consistent with the XRD results. Analysis of the Ir-*L*_3_-edge XANES spectra revealed that the white line maximum of Ir_ads_-NiO and Ir_emb_-NiO were located between those of Ir metal and IrO_2_, indicative of Ir valence states ca. +3 for Ir_ads_-NiO and Ir_emb_-NiO (Figures 3a and S7). Moreover, the higher white line intensity observed in Ir_ads_-NiO and Ir_emb_-NiO compared to IrO_2_ implies that these atomic Ir centers are clearly distinct from that of pristine IrO_2_ and suggest either the presence of emptier valence d-orbital states (d holes) and/or changes in the covalency respect with IrO_2_ (inset of Figure 3a).^23^

The local coordination environment of Ir single atoms was further analyzed by Ir *L*_3_-edge FT-EXAFS. The Ir *L*_3_-edge FT-EXAFS spectra of Ir_ads_-NiO and Ir_emb_-NiO were significantly different from IrO_2_ displaying broader peaks that suggest a certain degree of deviation from the ideal *Oh* geometry of IrO_2_ and indicate a different nature of the Ir sites (Figure 3b). Thus, both Ir_ads_-NiO and Ir_emb_-NiO showed a fitted distance of ∼2.0 Å for the first shell, which was attributed to the Ir–O distance with an overall coordination number (CN) of 6, expected for Ir sites (Figures 3b, S6b, and Table S3). A split first shell model was used, which although obtained Δ*R* = 0.15 Å for the fitted distances, which is at the limit of the resolution (Δ*R* = π/2Δ*k*, 0.15 Å), fits better the experimental spectra in comparison to a single path first shell model (Figure S8a,b and Table S4). Notably, a clear difference in the second shell was observed between the prepared materials and pristine IrO_2_. While IrO_2_ showed its characteristic scattering with remote Ir atoms at ∼3.1 to 3.5 Å, Ir_ads_-NiO and Ir_emb_-NiO displayed the absence of those features including the absence of direct Ir–Ir bonds, further confirming the atomic dispersion of Ir atoms and dismissing the presence of any Ir-based oxides nanoparticles. Instead, both Ir_ads_-NiO and Ir_emb_-NiO displayed an intense peak at ∼3.0 Å, typical for Ni–Ni distances in NiO. According to the Ir *L*_3_-edge wavelet transform EXAFS (WT-EXAFS) analysis, the contribution to this feature is far from the Ir–Ir interactions expected for Ir(0) and IrO_2_ and is consistent with the scattering of the absorbing Ir atoms with remote Ni centers, as suggested by the similar *k*/*R*-space relationship in the WT-EXAFS spectrum of pristine NiO (Figures 3c and S9). Thus, this further supports the stabilization of single Ir atoms on the NiO surface. Notably, the relative intensity of the assigned Ir–Ni distances at ∼3.0 Å respect with the first shell (Ir–O at ∼2.0 Å) of Ir_emb_-NiO increases in comparison to that of Ir_ads_-NiO. Thus, suggesting a higher Ir–Ni coordination number for Ir_emb_-NiO. FT-EXAFS fittings of the experimental Ir *L*_3_-edge spectra resulted in an Ir–Ni coordination number (CN) of ca. 3.5 for Ir_ads_-NiO, expected for a majority of surface adsorbed metal ion on the NiO substrate (Figure 3b and Table S3). In contrast, a higher Ir–Ni coordination number of 8 was found for Ir_emb_-NiO, suggesting that Ir sites are partially embedded within the NiO surface with a modulated second coordination sphere. For comparison purposes, an Ir-substituted NiO was prepared by introducing the Ir-precursor into the media during the electrodeposition of the NiO, instead of postsynthetic treatment, yielding (Ni_0.98_Ir_0.02_O). In contrast to Ir_ads_-NiO and Ir_emb_-NiO, the Ir in Ni_0.98_Ir_0.02_O is homogeneously distributed inside the bulk material, as evidenced by the XRD analysis, EDS mapping, and also by the similar Ir-loading found in ICP-OES, but the lower Ir/Ni ratio observed by surface-sensitive XPS techniques (see experimental section for details, Figure S10). A comparison of FT-EXAFS analysis for these samples (Figure S11) evidence a clear increasing trend in the intensity of the Ir–Ni peak, suggestive of a higher coordination number for Ni_0.98_Ir_0.02_O. The fitting of the later (Ni_0.98_Ir_0.02_O) indicates a coordination number of 11 for the Ir–Ni scattering in the second shell, indicative of a more homogeneous distribution of the Ir atoms in the bulk NiO (CN = 12 is expected for an inner Ni atom in NiO, Figure S8c and Table S3). Thus, it is proposed that the presented synthetic approaches can effectively modulate the local coordination environment of Ir single atoms in Ir_ads_-NiO, Ir_emb_-NiO, and Ni_0.98_Ir_0.02_O.

The valence states of the surface metal species were further assessed by XPS measurements.^24^ High-resolution Ir 4*f* XPS spectra of both Ir_ads_-NiO and Ir_emb_-NiO revealed partial positive oxidation states of atomic Ir atoms positioned below +4, consistent with the findings from Ir *L*_3_-edge XANES analysis (Figure 3d). As displayed in the high-resolution Ni 2*p*_3/2_ XPS spectra of Ir_emb_-NiO (Figure S12a,b), a notably higher ratio between the second and first peak compared to pristine NiO and Ir_ads_-NiO, suggested a significant increase in the valence states of surface Ni atoms, indicative of the enhanced electronic coupling between the partially encapsulated Ir and surface Ni atoms, driven by the increased Ir–Ni coordination. Conversely, the O 1*s* XPS spectra of both Ir_ads_-NiO and Ir_emb_-NiO displayed minimal changes (Figure S12b). Integration of both structural and spectroscopic characterizations demonstrates the successful regulation of the local coordination spheres of Ir single-atoms at the atomic interface by precisely modulating their steric locations on the NiO surface without altering the supports.

### 2.3 Electrocatalytic Performance toward OER

**Figure 4:**
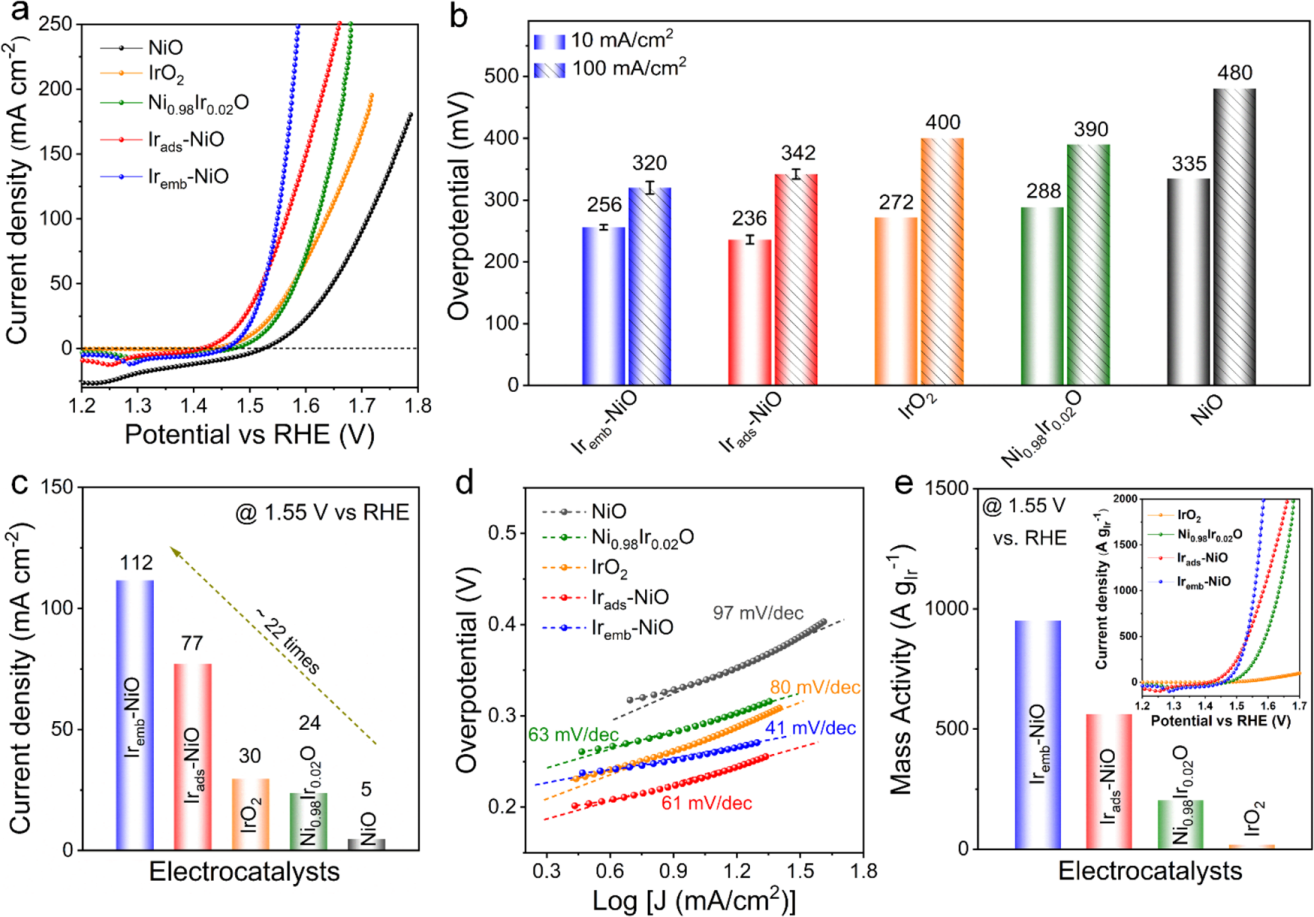
Electrocatalytic performances toward the OER. (a) OER LSV polarization curves (back-scanned from positive to negative potential), (b) the overpotentials required to reach 10 and 100 mA/cm^2^, (c) current density reached at 1.55 V vs RHE, and (d) corresponding Tafel plots in 1 M KOH. (e) Mass activity of Ir_ads_-NiO and Ir_emb_-NiO with control samples in 1 M KOH at 1.55 V vs RHE. The inset shows the noble-metal-mass-normalized OER LSV polarization curves of Ir_ads_-NiO and Ir_emb_-NiO with control samples in 1 M KOH.

In order to establish the correlation between the catalytic performance and specific steric effects, the electrocatalytic OER performances of Ir_emb_-NiO and Ir_ads_-NiO samples were assessed in alkaline media (1 M KOH) using a conventional three-electrode setup.^25^ The potential of the Hg/HgO reference electrode was calibrated in a hydrogen-saturated electrolyte, and all of the potentials were converted to the reversible hydrogen electrode (RHE) scale (Figure S13).^10^ The *iR*-compensated linear sweep voltammetry (LSV) polarization curves revealed that Ir_emb_-NiO exhibited excellent alkaline OER kinetics, requiring low overpotentials (η) of only 256 ± 4 and 320 ± 10 mV to reach current densities of 10 and 100 mA/cm^2^, respectively (Figure 4a,b). Notably, the OER performance of Ir_emb_-NiO surpassed that of state-of-the-art IrO_2_ (η_10_: 272 mV and η_100_: 400 mV), conventional Ir_ads_-NiO (η_10_: 236 ± 7 mV and η_100_: 342 ± 8 mV), Ni_0.98_Ir_0.02_O (η_10_: 288 mV and η_100_: 390 mV), and other control samples (Figure S14). As shown in Figure S14a,b, the substantial decrease in both the Ni^2+^/Ni^3+^ peak area and the peak potential to lower values upon Ir stabilization further substantiates the strengthened metal–support interaction between the surface Ir atoms and the NiO support. Moreover, Ir_emb_-NiO exhibited slightly higher onset potential compared to Ir_ads_-NiO, likely due to the partial encapsulation of Ir atoms. Furthermore, Ir_emb_-NiO achieved the highest current density at 1.55 V vs RHE, which was ∼1.5, 3.7, and 22 times higher than Ir_ads_-NiO, IrO_2_, and pristine NiO, respectively (Figure 4c). The Ir_emb_-NiO manifests an impressively small Tafel slope of 41 mV/dec, in contrast to Ir_ads_-NiO (61 mV/dec) and IrO_2_ (80 mV/dec), highlighting its superior reaction kinetics (Figure 4d). Furthermore, the Ir_emb_-NiO delivered an ultrahigh noble-metal-mass-normalized alkaline OER activity compared to IrO_2_ and Ir_ads_-NiO, indicating high atom utilization and remarkable economic efficiency toward OER (Figure 4e). The superior alkaline OER activity of Ir_emb_-NiO was further corroborated by its smallest charge transfer resistance (*R*_CT_) coupled with a higher electrochemically active surface area (ECSA), suggesting rapid electron transfer kinetics with a high abundance of active sites, thereby enhancing the OER performance (Figures S15 and S16). The excellent intrinsic activity of Ir_emb_-NiO over Ir_ads_-NiO was further corroborated by the ECSA-normalized alkaline OER LSV polarization curve (Figure S17).

**Figure 5:**
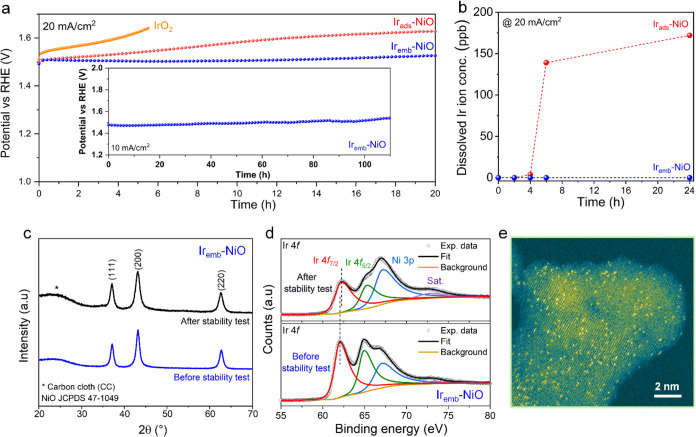
Stability test and poststability characterizations. (a) Chronopotentiometric stability test of Ir_ads_-NiO, Ir_emb_-NiO, and IrO_2_, in 1 M KOH at a current density of 20 mA/cm^2^. The inset shows the long-term stability test of Ir_emb_-NiO in 1 M KOH at a current density of 10 mA/cm^2^. (b) Dissolved Ir-ion concentrations measured for Ir_ads_-NiO and Ir_emb_-NiO in the electrolyte by ICP-OES. (c) XRD pattern and (d) high-resolution Ir 4f XPS spectra of Ir_emb_-NiO before and after the stability test for 20 h. (e) AC-HAADF STEM image of Ir_emb_-NiO after the stability test for 20 h.

Beyond superior activity, the long-term durability of the single-atom catalyst is of widespread concern due to the low coordination of single atoms (weak metal–support interaction) and harsh oxidative operating conditions.^12^ The durability assessments of catalysts were conducted by chronopotentiometry analysis (Figure 5a).^26,27^ The Ir_emb_-NiO with enhanced Ir–Ni second-shell interaction displayed excellent stability for 20 h at 20 mA/cm^2^ during the alkaline OER, outperforming both commercial IrO_2_ and conventional Ir single-atoms adsorbed on NiO (Ir_ads_-NiO). Remarkably, Ir_emb_-NiO displayed an ultralow degradation rate of ∼1 mV/h, significantly outperforming Ir_ads_-NiO (∼7 mV/h) and IrO_2_ (∼26 mV/h) by 7 and 26 times, respectively. Moreover, the superior durability of Ir_emb_-NiO over Ir_ads_-NiO was verified by monitoring dissolved Ir-ions in the electrolyte during electrolysis using ICP-OES (Figures 5b and S18). The Ir_emb_-NiO demonstrated negligible leaching of atomic Ir atoms, contrasting with conventional Ir_ads_-NiO, which exhibited substantial Ir single-atom leaching due to their weak metal–support interactions during the durability test. This underscores the significant advantage of higher Ir–Ni second-shell coordination in Ir_emb_-NiO, providing structural stability to the atomic Ir sites compared to that of conventional Ir_ads_-NiO with lower second-shell coordination. The superimposed LSV curves obtained before and after the durability test in alkaline media provide additional evidence for the preservation of active sites alongside sustained high catalytic activity (Figure S19).

Poststability characterizations including XRD, XPS, HAADF-STEM, FE-SEM, XAS, and ICP-OES of Ir_emb_-NiO revealed that the crystal structure, morphology, chemical states of Ir/Ni, and the atomic dispersion of Ir single atoms with higher second-shell coordination were well-maintained, reaffirming the structural robustness of Ir_emb_-NiO (Figures 5c–e, S20, S21 and Table S3). Poststability EDS analysis indicated a comparable Fe uptake (<0.2 wt %) from the electrolyte in both Ir_emb_-NiO and Ir_ads_-NiO, excluding the influence of Fe impurities on the enhanced stability observed in Ir_emb_-NiO (Figure S22). Moreover, Ir_emb_-NiO also exhibited sustained long-term durability for at least 110 h at 10 mA/cm^2^ in alkaline conditions without any noticeable degradation, further highlighting its potential suitability for practical applications (inset of Figure 5a and Table S5). In addition to long-term durability, high energy conversion efficiency is a critical benchmark for an efficient electrocatalyst. The Ir_emb_-NiO demonstrated a high energy-conversion efficiency (Faradaic efficiency) of ∼94.8 ± 1.6% for the alkaline OER, indicating that nearly all of the charge is utilized for the OER without any associated parasitic reactions (Figure S23). The commendable activity and enhanced durability demonstrated by Ir_emb_-NiO were comparable and ever better than most of the recently reported alkaline OER electrocatalysts (Figures S24 and S25).

### 2.4 *Operando* Characterizations of the Structure and Mechanistic Investigations

**Figure 6:**
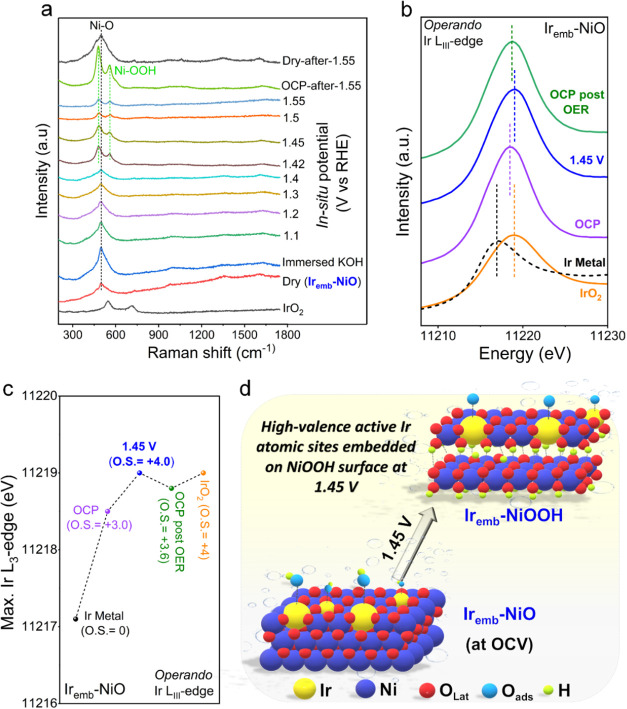
In situ Raman spectroscopy and *operando* X-ray absorption spectroscopy characterizations. (a) In situ Raman spectroscopy measurements for Ir_emb_-NiO recorded during alkaline OER from 1.1 to 1.55 V vs RHE. (b) Experimental Ir-*L*_3_ edge operando XANES spectra of Ir_emb_-NiO at OCP, 1.45 V, and OCP post OER with Ir metal and IrO_2_ reference sample. (c) Positions of Ir-*L*_3_ edge maximums at applied potentials and their correlation with the iridium oxidation state for Ir_emb_-NiO. (d) Schematic illustration of the structure reconstruction process for Ir_emb_-NiO under an applied potential of 1.45 V vs RHE.

To gain further insights into the reality-close active structure of Ir_emb_-NiO under operating conditions and to elucidate the reaction mechanism, we employed in situ Raman spectroscopy, *operando* X-ray absorption spectroscopy, pH-dependent activity measurements, and a molecular probe to diagnose a specific OER intermediate. The in situ Raman spectra of Ir_emb_-NiO revealed that under the applied oxidative potential, two bands gradually emerged at ∼483 and 559 cm^–1^, which were assigned to the E_g_ bending vibration (Ni^3+^-O) and A_1g_ stretching vibration (Ni^3+^-O) mode of nickel (Oxy)hydroxide (NiOOH), respectively (Figure 6a).^28^ Notably, the emergence of Raman bands for Ni^3+^-O commenced at a significantly lower potential (1.42 V vs RHE) for Ir_emb_-NiO compared to pristine NiO, attributed to the enhanced metal–support interaction between the Ir single atom and NiO support in Ir_emb_-NiO (Figure S26).

The preceding electrochemical analysis indicated that the stabilized atomic Ir sites predominantly contributed to enhanced OER activity. Hence, *operando* Ir *L*_3_-edge XAS analysis of Ir_emb_-NiO was performed to investigate the potential-dependent oxidation of Ir single-atoms under the OER operating conditions (Figures 6b,c and S27). Analysis of the maximum edge position of Ir *L*_3_-edge XANES spectra at the open circuit potential (OCP) revealed an Ir valence state close to +3, in agreement with the analyzed ex situ sample (Figure 6c).^23^ Under the applied OER potential of 1.45 V versus RHE, the maximum edge position exhibited a significant shift compared to that of the OCP, indicating the formation of species with a higher oxidation degree under catalytic conditions. The correlation between the edge position and the oxidation state (O.S.) suggests an increase of Ir valence state from Ir^3+^ at OCP (5d^6^) to high-valence Ir^4+^ (5d^5^), indicating that the higher Ir oxidation states should be most likely stabilized by an oxo ligand (O*).^23,29,30^ Thus, the increase in the oxidation of atomic Ir sites under applied OER potential is typically associated with the deprotonation of the Ir–OH* to form active Ir–O* via proton-coupled electron transfer, where the resting state (Ir–O*) likely participated in the rate-determining step of OER.^30^ Furthermore, the active high-valence atomic Ir atoms predominantly retained their high chemical states, even when the applied potential was reversed to the OCP, indicating that the valence change was partially reversible. The above in situ Raman and *operando* XAS analysis collectively demonstrated that the Ir_emb_-NiO underwent obvious structure reconstruction to generate high-valence atomic Ir atoms embedded on the NiOOH surface, where the electrochemically formed active high-valence Ir sites could facilitate O–O formation for achieving superior OER performance.^30,31^Figure 6d schematically illustrates the expected structure reconstruction process of Ir_emb_-NiO to high-valence Ir single atoms on the NiOOH surface (Ir_emb_-NiOOH) under the applied OER potential of 1.45 V vs RHE.

In alkaline OER, two primary mechanisms have been extensively studied: the adsorbate evolution mechanism (AEM) and the lattice oxygen mechanism (LOM).^32^ AEM operates at single metal catalytic sites, involving the simultaneous transfer of proton–electrons and displaying a linear scaling relationship, resulting in a high theoretical overpotential due to the adsorption of multiple intermediates (*OH, *O, and *OOH). In contrast, LOM, also occurring at individual catalytic site, involves the participation of lattice oxygen to form molecular O_2_ via direct O–O_lattice_ coupling, bypassing the OOH* intermediate and breaking the linear scaling relationship of AEM, thereby promoting more favorable OER kinetics.^33^ However, the LOM pathway could significantly destabilize the single metal atoms’ first coordination sphere due to the cleavage of metal-O_lattice_ bonds to facilitate lattice oxygen participation in the OER, potentially compromising the long-term stability of conventional SACs.

**Figure 7:**
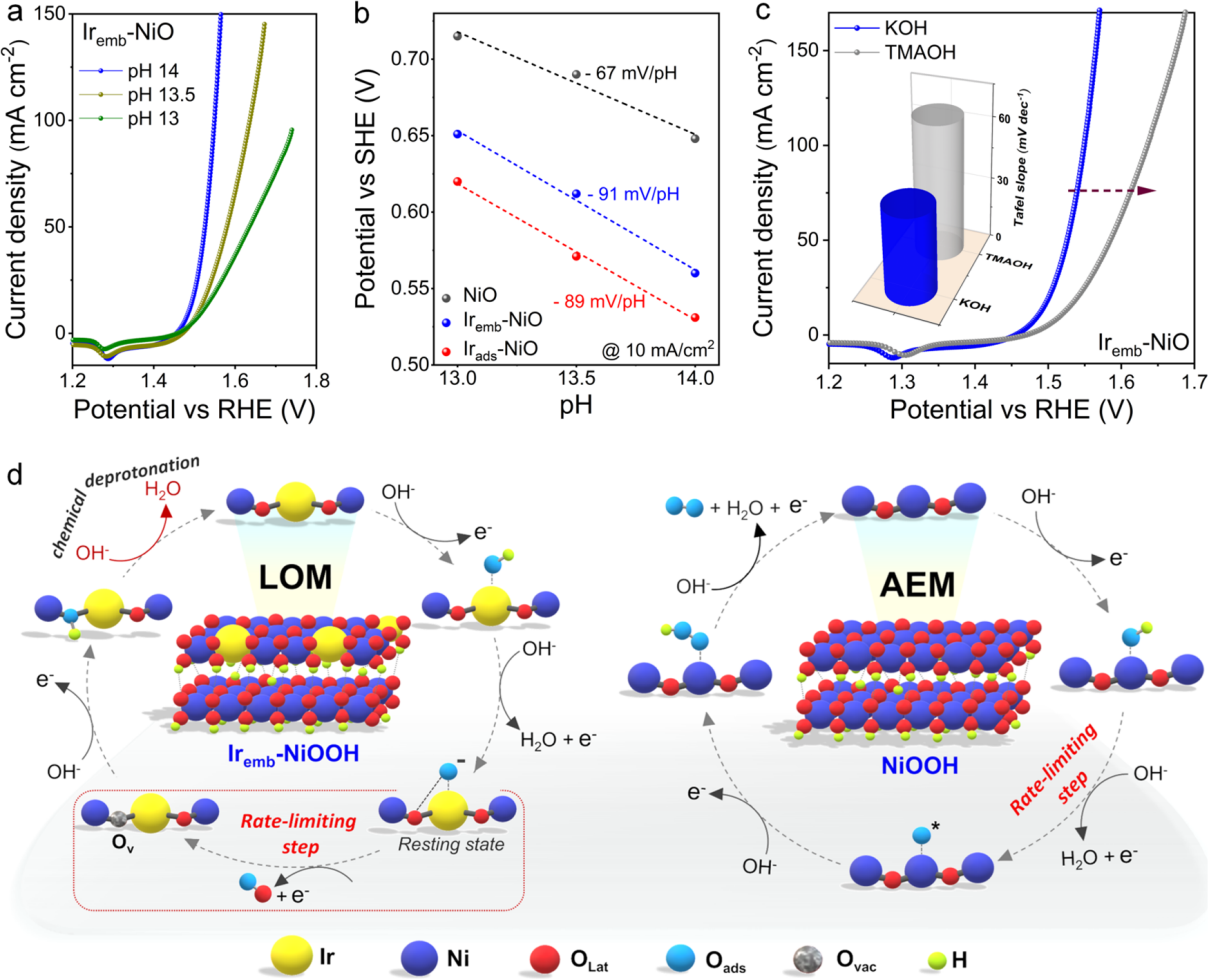
Mechanistic investigations. (a) pH-dependent LSV polarization curves of Ir_emb_-NiO recorded in KOH. (b) pH-dependence of the OER potential on the SHE scale at 10 mA/cm^2^ for Ir_emb_-NiO, Ir_ads_-NiO, and NiO. (c) LSV polarization curves of Ir_emb_-NiO recorded in 1 M KOH and tetramethylammonium hydroxide (TMAOH). The inset shows the Tafel slope comparison of Ir_emb_-NiO in 1 M KOH and TMAOH. (d) Proposed schematic illustration of the lattice oxygen mechanism (LOM) process for Ir_emb_-NiO (reconstructed to Ir_emb_-NiOOH) and the adsorbate evolution mechanism (AEM) process for NiO (reconstructed to NiOOH).

To elucidate whether Ir_emb_-NiO, Ir_ads_-NiO, and NiO adhere to the LOM or AEM pathway during alkaline OER, we conducted OER LSV measurements at different pH conditions (pH: 14, 13.5, and 13). The pH-dependent OER activity, indicative of proton transfer decoupled from the electron transfer process, offers insights into the LOM pathway, which entails lattice oxygen participation, refilling of generated oxygen vacancies via OH^–^ ions from the electrolyte, and subsequent chemical deprotonation steps.^34^ As depicted in Figures 7a and S28, the catalytic activities of both samples containing Ir single-atoms (Ir_emb_-NiO and Ir_ads_-NiO) demonstrated strong pH-dependent OER kinetics on the RHE scale, suggesting the involvement of nonconcerted proton–electron transfer steps or LOM pathway for OER.^34,35^ Conversely, pristine NiO exhibited pH-independent OER kinetics on the RHE scale, typical of the AEM pathway.^36^ Moreover, the potential versus standard hydrogen electrode (SHE) at 10 mA/cm^2^ was plotted against the solution pH to generate the potential-pH diagram of water (Figure 7b). The slope of ∼−90 mV/pH for both Ir_emb_-NiO and Ir_ads_-NiO deviates significantly from the expected −59 mV/pH (Nernstian potential shift) for the 1 e^–^/1 H^+^ process, further indicating the decoupling of proton transfer from electron transfer and the OER at atomic Ir sites followed the LOM pathway.^37−39^ In contrast, the slope of −67 mV/pH for pristine NiO, closer to the −59 mV/pH (Nernstian potential shift), suggested a proton-coupled electron transfer process, indicative of the conventional AEM pathway strictly followed at Ni sites.^39^ Consequently, the integration of active high-valence Ir single-atoms on the NiO surface could trigger neighboring lattice oxygen participation and induce the switch of the OER mechanism.

One characteristic feature of LOM involves the binding of adsorbed oxygen intermediates to lattice oxygen on the catalyst surface, forming peroxo-like O_2_^2–^ negatively charged oxygenated species.^34^ To monitor these charged intermediates during the OER process, tetramethylammonium cation (TMA^+^) was utilized as a chemical probe, known for its specific interaction with negatively charged species and inhibiting the LOM cascade.^35^ As revealed in Figures 7c and S29, both Ir_emb_-NiO and Ir_ads_-NiO displayed reduced OER activity and increased Tafel slopes in the presence of tetramethylammonium hydroxide (TMAOH), indicating partial inhibition of the LOM pathway due to the strong electrostatic interaction between TMA^+^ and negative oxygenated intermediates. Conversely, negligible changes in the OER kinetics observed in KOH and TMAOH for NiO further validated the conventional AEM pathway. The above analysis including ex situ*/operando* characterizations and electrochemical tests indicates that the Ir_emb_-NiO, featuring high-valence Ir sites under operational condition, adheres to the LOM pathway for OER, thus overcoming the theoretical limit of the conventional AEM and yields superior OER kinetics along with sustained durability, thanks to the modulated local coordination sphere.

Based on the above findings, we propose the LOM pathway for Ir_emb_-NiO (reconstructed to Ir_emb_-NiOOH) and the AEM pathway for NiO (reconstructed to NiOOH), schematically illustrated in Figure 7d. The LOM pathway initiates with the adsorption of the OH^–^ ion from the electrolyte onto the atomic Ir site, followed by the deprotonation of Ir–OH* to the Ir–O* resting state. The adsorbed-O on the high-valence Ir site undergoes direct coupling with the neighboring lattice-O to release molecular oxygen (rate-determining step), involving only e- transfer. Subsequently, the generated oxygen vacancy is refilled with OH^–^ from the electrolyte, followed by the chemical deprotonation of OH*, which involves only H^+^ transfer, thereby regenerating the active site. The AEM pathway for pure NiOOH commences with the adsorption of OH* on the Ni site. Then, Ni–OH* undergoes deprotonation to form Ni–O* (rate-determining step),^40^ followed by a nucleophilic attack from OH^–^ to form NiOOH* intermediate. Ultimately, the OOH* intermediate is converted to molecular oxygen assisted by OH^–^ from the electrolyte, thus completing the pathway.

## 3 Conclusions

We have successfully modulated the local coordination spheres of the Ir single-atoms at the atomic-interface by precisely adjusting their spatial localization on the NiO surface, resulting in outstanding alkaline OER kinetics and long-term stability. Comparative analysis with conventional Ir_ads_-NiO revealed that the Ir_emb_-NiO showcases a 2-fold increase in Ir–Ni second-shell coordination, as confirmed by Ir *L*_3_-edge XAS analysis, indicating enhanced metal–support interactions and precise control over the microenvironments of Ir single-atoms. The Ir_emb_-NiO demonstrated superior alkaline OER kinetics with high mass activity and sustained operation for more than 110 h, outperforming both IrO_2_ and Ir_ads_-NiO. Furthermore, the Ir_emb_-NiO reconstructed to Ir_emb_-NiOOH during OER, featuring high-valence active Ir atomic-sites triggers lattice oxygen participation via LOM pathway rather than traditional AEM, revealed by in situ Raman spectroscopy, *operando* XAS analysis and pH-dependent activity test. Moreover, the enhanced Ir–Ni second-shell coordination in Ir_emb_-NiO conferred supplementary structural stability to surface atomic Ir sites, alleviating their dissolution and ensuring superior alkaline OER kinetics while maintaining excellent stability. This work offers both fundamental and technological insights for the development of highly active and stable single-atom catalysts through precise regulation of their local coordination environments, particularly for alkaline water electrolysis and future renewable energy conversion devices.

## 4 Experimental Methods

### 4.1 Chemicals

Nickel(II) nitrate hexahydrate (Ni(NO_3_)_2_·6H_2_O; Sigma-Aldrich, ≥99%), hexachloroiridium acid hydrate (H_2_IrCl_6_·xH_2_O; Sigma-Aldrich, ≥99.9%), potassium nitrate (KNO_3_; Sigma-Aldrich, ≥ 99%), tetramethylammonium hydroxide solution (((CH_3_)_4_N(OH)); Sigma-Aldrich, 25 wt % in H_2_O), potassium hydroxide (KOH; Sigma-Aldrich, ≥85%), nitric acid (HNO_3_; Sigma-Aldrich, 70%), ethanol (C_2_H_5_OH; Sigma-Aldrich, ≥99.9%), Toray carbon cloth (CC, Alfa Aesar), and the nafion perfluorinated resin solution (5 wt %, Sigma-Aldrich) were used without further purification.

### 4.2 Synthesis of Ni-Layered Double Hydroxide (Ni-LDH) Support

In a typical protocol, a CC substrate underwent a thorough cleansing process with nitric acid, followed by multiple rinses with deionized water and ethanol under ultrasonication. Subsequently, the CC was dried completely before the electrodeposition of Ni-LDH precursors onto its surface. This electrodeposition process occurred at −1.3 V vs Ag/AgCl for 10 min in an aqueous solution containing 0.1 M Ni(NO_3_)_2_·6H_2_O using a typical three-electrode electrochemical workstation. The resulting samples were subsequently washed with deionized water and ethanol and then dried at 60 °C overnight.

### 4.3 Synthesis of NiO Support

To obtain NiO support, the synthesized Ni-LDH underwent calcination at 350 °C for 3 h at a heating rate of 3 °C min^–1^, followed by natural cooling to room temperature.

### 4.4 Synthesis of Ir_ads_-NiO

In a standard procedure, the NiO support was immersed in an ethanolic solution containing H_2_IrCl_6_·xH_2_O (6 mg/mL) for 30 min, followed by drying in an oven. Subsequently, the sample underwent annealing in air at 350 °C for 2 h with a heating rate of 5 °C min^–1^ (sample loading: 2 mg/cm^2^) to obtain Ir_ads_-NiO. For control samples, Ir_ads_-NiO was also synthesized by varying the ethanol Ir-salt solution (3 and 10 mg/mL) denoted as Ir_ads_-NiO (3 mg/mL) and Ir_ads_-NiO (10 mg/mL).

### 4.5 Synthesis of Ir_emb_-NiO

For the synthesis of Ir_emb_-NiO, the Ni-LDH support was directly immersed in an ethanolic solution containing H_2_IrCl_6_·xH_2_O (6 mg/mL) for 30 min, followed by drying in an oven. Subsequently, the samples were calcined in air at 350 °C for 3 h with a heating rate of 3 °C min^–1^ (sample loading: 2 mg/cm^2^) to obtain Ir_emb_-NiO.

### 4.6 Synthesis of Ir-Substituted NiO (Ni_0.98_Ir_0.02_O)

To synthesize Ni_0.98_Ir_0.02_O, the Ir-substituted Ni-LDH was electrodeposited onto the CC at −1.3 V vs Ag/AgCl for 10 min in an aqueous solution containing an appropriate mixture of 0.1 M Ni(NO_3_)_2_·6H_2_O and 0.1 M H_2_IrCl_6_·xH_2_O using a typical three-electrode electrochemical workstation, followed by calcination in air at 350 °C for 3 h at a heating rate of 3 °C min^–1^.

### 4.7 Material Characterization

The crystal structures and orientations of all catalysts were analyzed by using various techniques. X-ray diffraction (XRD) measurements were conducted at room temperature using a Stoe theta/theta diffractometer operating in transmission mode, following Bragg–Brentano geometry with a Cu Kα_1,2_ radiation X-ray source (λ: 1.5406 Å). FE-SEM images were acquired by using a JEOL 7500F FE-SEM. EDS patterns were collected using a Hitachi TM-3030 microscope equipped with a Si(Li) Pentafet plus detector from Oxford instruments. The ICP-OES measurements were performed using a SPECTROGREEN instrument, with electrolyte solution samples extracted from the electrochemical cell before and after the reaction. Aberration-corrected HAADF-STEM images were obtained on a JEOL JEM ARM 200F instrument at 200 kV. XPS analysis was carried out using a VG ESCALAB 220i-XL with an X-ray source employing monochromatic Al Kα anode (1486.6 eV), operated at 200 W and 15 kV. The analysis chamber maintained a base pressure of 5 × 10^–10^ mbar. The C 1s peak for contaminant carbon was used as a reference at 284.5 eV for correction of the binding energy for surface charging.

#### 4.7.1 XAS Measurements

##### 4.7.1.1 Ex Situ Measurements

The Ni *K*-edge and Ir *L*_3_-edge were utilized to conduct measurements on NiO, Ir_ads_-NiO, Ir_emb_-NiO, and Ni_0.98_Ir_0.02_O in fluorescence mode, while the reference samples metallic Ni, NiO, metallic Ir, and IrO_2_ were analyzed in transmission mode. Spectra were acquired at the BL10C beamline of the Pohang Light Source (PLS-II, Korea). Powder samples were prepared by diluting the corresponding powder with cellulose in a pellet (φ = 6 mm), followed by placement in the sample holder and sealing with 30 μm of Kapton tape. The monochromatic X-ray beam was generated using a liquid-nitrogen-cooled Si (111) double-crystal monochromator (Bruker ASC) sourced from high-intensity X-ray photons of a multipole wiggler source. X-ray absorption spectroscopic data were recorded in fluorescence mode using 7 channels silicon drift detectors (SDD, Rayspec Ltd.), which offer high efficiency for extremely low concentration elements. Higher order harmonic contaminations were eliminated by detuning to reduce the incident X-ray intensity by ∼30%. Energy calibration was performed concurrently for each measurement with each reference metal placed in front of the third ion chamber. Incident energy was calibrated by referencing the inflection point of the corresponding metallic foil, which was 8333.0 eV for Ni and 11215 eV for Ir. The final spectra were processed and normalized using the Athena and Artemis program included in the DEMETER package.^41^

### 4.8 *Operando* X-Ray Absorption Spectroscopy

*Operando* measurements were conducted to monitor the alterations in the Ir *L*_3_-edge for the Ir_emb_-NiO sample. The Ir *L*_3_-edge XAS measurements were executed at the SAMBA beamline of the SOLEIL synchrotron, operating with an electron beam current of 450 mA. The incident energy was selected by a Si (200) double crystal monochromator. Incident flux was ca. 1 × 10^10^ ph/s using a beam size of 4 × 0.5 mm. Fluorescence spectra were captured using a 36-element germanium detector. Ir_emb_-NiO electrodes were situated within a custom-built spectroelectrochemical (SEC) flow cell, and the SEC cell was affixed to the sample stage using a custom-designed plate intended to prevent and contain any electrolyte leaks. Throughout the measurement, the electrolyte (1 M KOH) was continuously circulated through the cell (1.5–2.0 mL/min) using a membrane pump (Flow unit, Fluigent) regulated by a standalone vacuum pressure-based controller (Fluigent) to refresh the solution and maintain a constant pH. The pump operated in push mode using Ar. A SP-300 potentiostat (Biologic) facilitated the execution of the cyclic voltammetry (CVs) and CPE experiments during the measurements. Typically, 2 repetitive CV cycles were performed before applying the desired potential to condition the electrode and estimate the potential applied during the CPE. Subsequently, controlled potential electrolysis was conducted at the desired potentials (1.45 V vs RHE), while simultaneous Ir *L*_3_-edge XAS measurements were conducted. Samples were maintained at room temperature, and no evidence of radiation-induced damage to the samples was observed during the measurements. The incident energy was calibrated by assigning the inflection point of an Ir foil to 11215 eV. Final spectra were processed and normalized using Athena and Artemis included in the DEMETER package.^41^

### 4.9 Electrochemical Measurements

The electrochemical assessments were conducted by utilizing a VSP-300 BioLogic potentiostat in a standard three-electrode setup with 1 M KOH as the electrolyte. The freshly prepared electrode served as the working electrode, while Hg/HgO (1 M KOH) functioned as the reference electrode, and Pt mesh served as the counter electrode. To ensure similar loadings of different catalysts on support, all of the synthesis parameters such as electrodeposition time, electrodeposition potential, concentration of metal precursor solution, electrodeposition area, and calcination temperature and time were almost kept the same during the synthesis process. The reference electrode potential was calibrated in H_2_-saturated 1 M KOH, and all potentials were subsequently converted to the reversible hydrogen electrode (RHE) scale using the equation
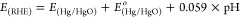
For the fabrication of the working electrode for commercial IrO_2_, 5 mg of catalyst powder was dispersed in 500 μL of ethanol containing 20 μL of 5% Nafion and sonicated for 60 min to achieve a homogeneous ink. Subsequently, a specific volume of the ink was drop-cast onto CC (loading: 2 mg cm^–2^) and allowed to dry under ambient conditions. Prior to electrochemical measurements, the electrodes underwent saturation via cyclic voltammetry (CV) scans at a scan rate of 100 mV s^–1^ in an argon-purged electrolyte. Linear sweep voltammetry (LSV) was conducted at a slow scan rate of 5 mV s^–1^ to minimize the capacitive contribution. Nyquist plots were obtained through electrochemical impedance spectroscopy measurements in the faradaic region to estimate the solution resistance (*R*_s_) and charge transfer resistance (*R*_CT_). The double-layer capacitance (*C*_dl_) was determined by collecting CVs at various scan rates (10, 15, 20, 25, and 30 mV s^–1^) in the nonfaradaic region. The electrochemically active surface area (ECSA) was calculated from the *C*_dl_ value using a specific capacitance of 0.06 mF/cm^2^.^42^ Long-term durability testing was conducted via chronopotentiometry at a constant current density. To measure the faradaic efficiency, the actual amount of gas (oxygen) produced was measured using the water displacement method in an airtight vessel.^10^ All potentials were *iR*-corrected with respect to the ohmic resistance of the solution obtained from the Nyquist plot unless stated otherwise
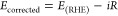
For the pH-dependent study, a series of KOH solutions were prepared with pH values of 14, 13.5, and 13. To maintain a constant ionic strength, an appropriate amount of KNO_3_ was added to the electrolyte.^43^

### 4.10 In Situ Raman Spectroscopy Measurement

The in situ Raman measurements were conducted by utilizing a customized in situ electrochemical flow cell in conjunction with an InVia Renishaw Raman microscope equipped with a 532 nm laser excitation wavelength and a 1800 l/mm grading, coupled with a 50× objective lens. The sample ink was drop-casted onto the surface of rough Au foil and served as the working electrode, while Pt wire and Hg/HgO were employed as the counter and reference electrodes, respectively. A 0.1 M KOH electrolyte with a pH of 13 was utilized. During the OER measurements, the flow rate of the electrolyte (5–8 mL/min) was controlled using a peristaltic pump. Ten consecutive scans were conducted with a 10-s exposure time at 0.5 mW laser power to obtain in situ Raman spectra. The in situ Raman spectra were recorded using chronoamperometric mode with the potential held for 3 min ranging from +1.1 to 1.55 V vs RHE.

## Supporting Information Available

Structural characterizations (FE-SEM, EDS, and HAADF-STEM), XANES, EXAFS, XPS, in situ Raman analysis, electrochemical measurements, measurements after catalysis (SEM, EDS, XPS, XANES, and EXAFS), and comparison plot (PDF)
