# Supplementary material for: Regulating Local Coordination Sphere of Ir Single Atoms at the Atomic Interface for Efficient Oxygen Evolution Reaction
Source: J Am Chem Soc. 2024 Oct 8;146(48):32953–64. doi: 10.1021/jacs.4c08847 (PMC11622227; doi:10.1021/jacs.4c08847)
Supplement: Supplementary file 1 — ja4c08847_si_001.pdf [file ja4c08847_si_001.pdf]

SUPPORTING INFORMATION

Regulating Local Coordination Sphere of Ir Single-Atoms at the Atomic-Interface for Efficient Oxygen Evolution Reaction

Ashwani Kumar[1], Marcos Gil-Sepulcre[2], Jean Pascal Fandré[1], Olaf Rüdiger[2], Min Gyu Kim[3], Serena DeBeer[2], Harun Tüysüz[*1, 4]

[1]Max-Planck-Institut für Kohlenforschung, 45470 Mülheim an der Ruhr, Germany

[2]Max Planck Institute for Chemical Energy Conversion, Stiftstrasse 34–36, D-45470 Mülheim an der Ruhr, Germany

[3]Beamline Research Division, Pohang Accelerator Laboratory (PAL), Pohang 790-784, South Korea

[4]IMDEA Materials Institute, Calle Eric Kandel 2, 28906, Getafe, Madrid, Spain

*Email of correspondence: tueysuez@kofo.mpg.de & harun.tuysuz@imdea.org

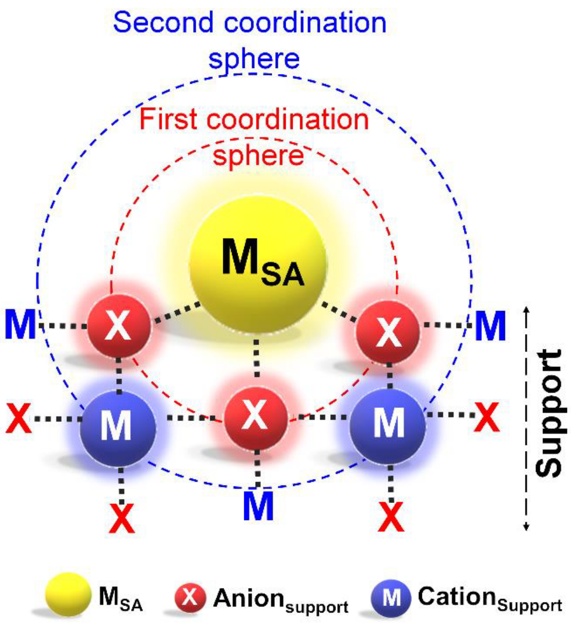

Figure S1. Schematic illustration of the local coordination spheres of metal single atoms (M_{SA}).

The first coordination sphere shares strong electronic hybridization with the metal single atom, which largely determines the oxidation state and intrinsic catalytic properties of SACs. While the second coordination sphere could provide additional structural stability to the single metal atom via the long-range interactions, which could enhance the stability of the SACs over long-term operations.

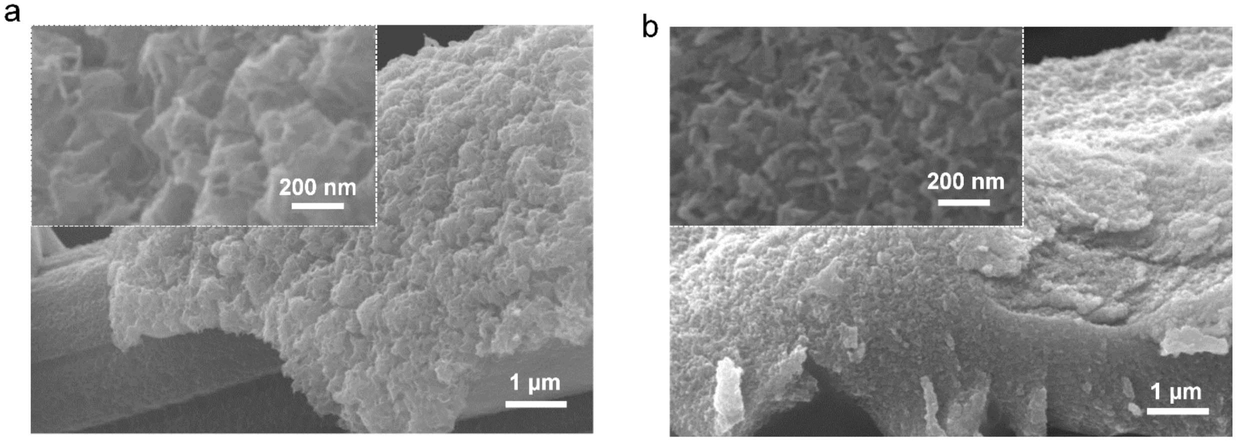

Figure S2. Field-emission scanning electron microscopy images of Ir_{ads}-NiO (a) and Ir_{emb}-NiO (b).

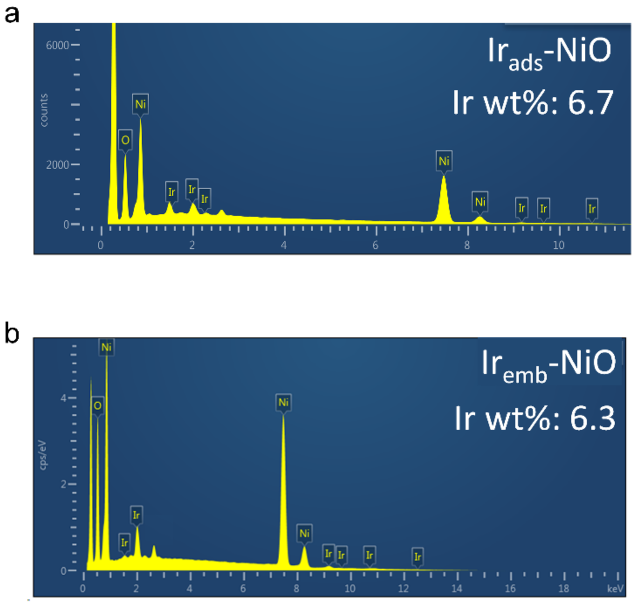

Figure S3. EDS pattern of Ir_{ads}-NiO (a) and Ir_{emb}-NiO (b).

Table S1. Weight % of metal loading obtained from EDS and ICP-OES analysis.

Sample	ICP-OES		Average Ir (wt%) from EDS and ICP-OES
	Ni (wt%)	(Ir wt%)	
Ir_{ads}-NiO (6 mg/mL, Ir-EtOH sol.)	93.7	6.3	6.5 ± 0.2
Ir_{emb}-NiO (6 mg/mL, Ir-EtOH sol.)	93.9	6.1	6.2 ± 0.1
Ir_{ads}-NiO (3 mg/mL, Ir-EtOH sol.)	94.5	5.5	-
Ir_{ads}-NiO (10 mg/mL, Ir-EtOH sol.)	91.2	8.8	-
Ir-doped NiO ($Ni_{0.98}Ir_{0.02}O$)	93.8	6.2	-

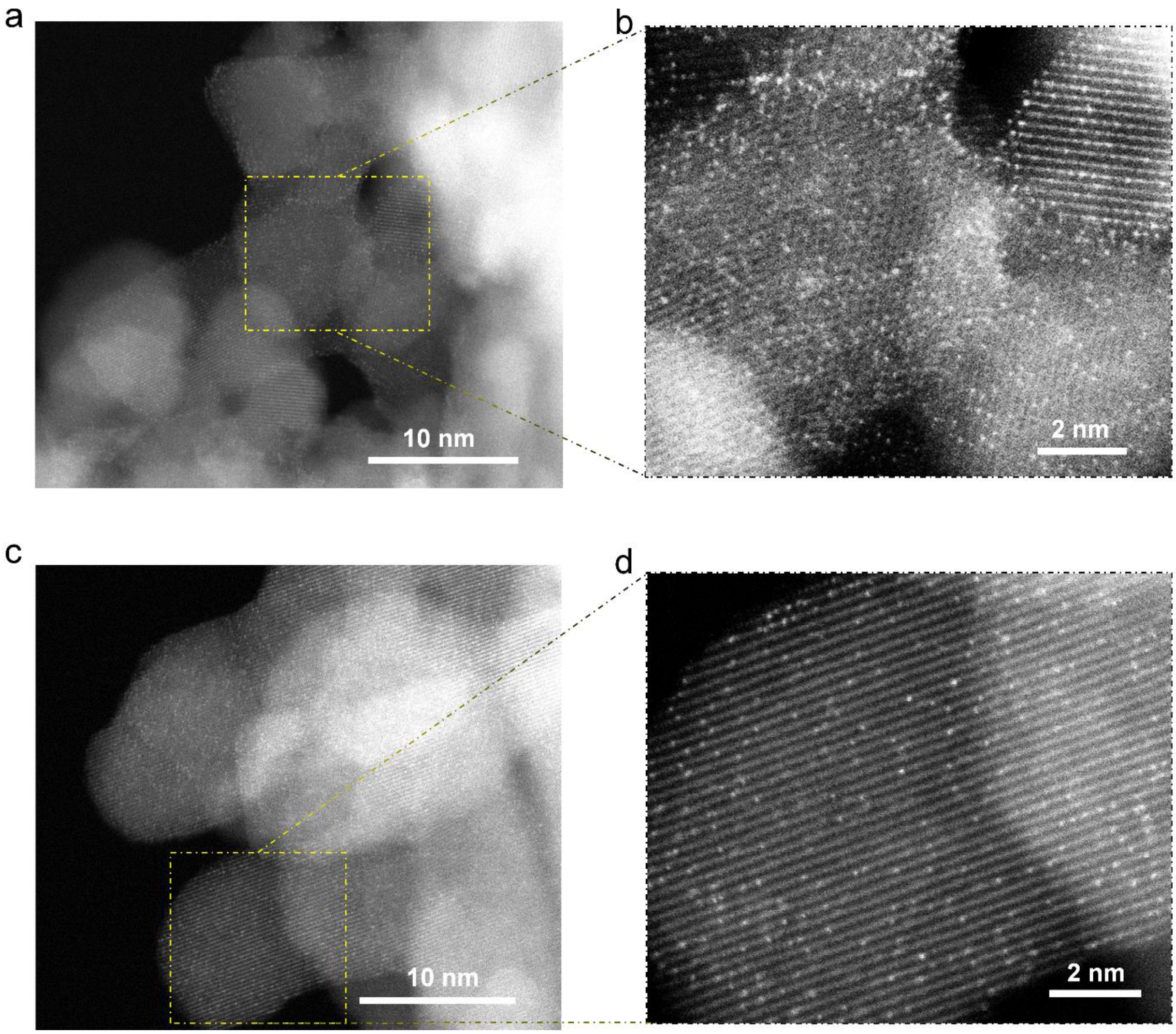

Figure S4. AC-HAADF-STEM images of Ir_{ads}-NiO at low-magnification (a), and high-magnification (b). AC-HAADF-STEM images of Ir_{emb}-NiO at low-magnification (c), and high-magnification (d).

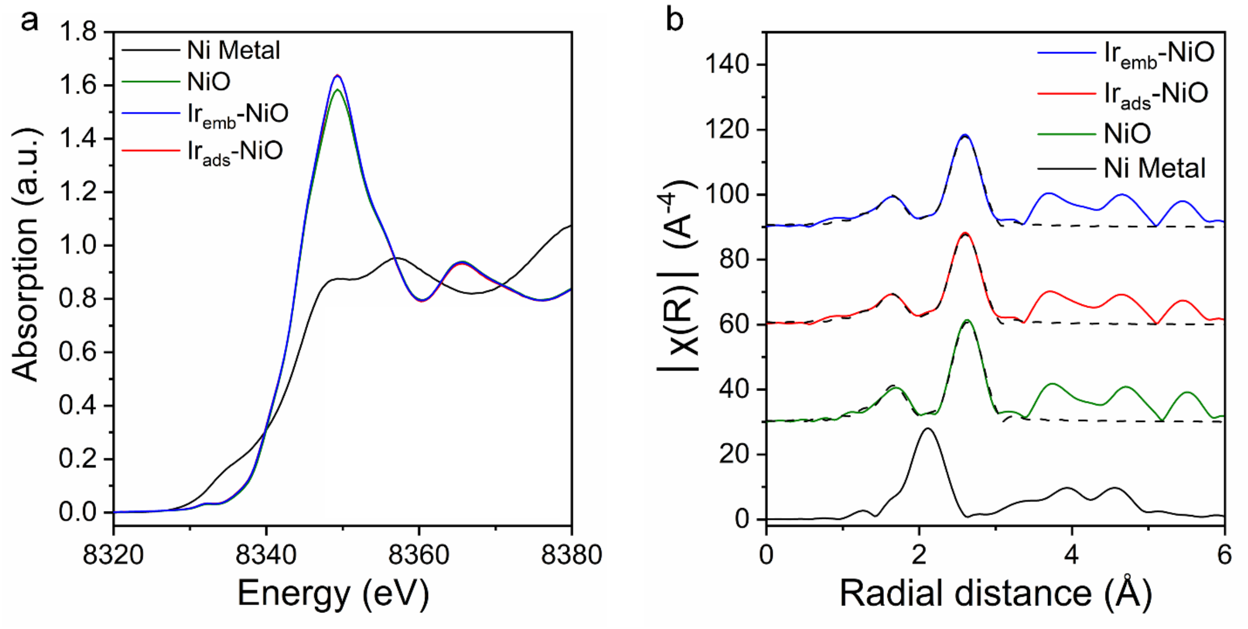

Figure S5. (a) Experimental Ni K_3-edge XANES spectra of Ir_{ads}-NiO and Ir_{emb}-NiO samples with Ni metal and NiO reference samples. (b) Experimental (solid line) and fitted (dashed line) Ni K-edge FT-EXAFS spectra of Ir_{ads}-NiO and Ir_{emb}-NiO samples with Ni metal and NiO reference samples.

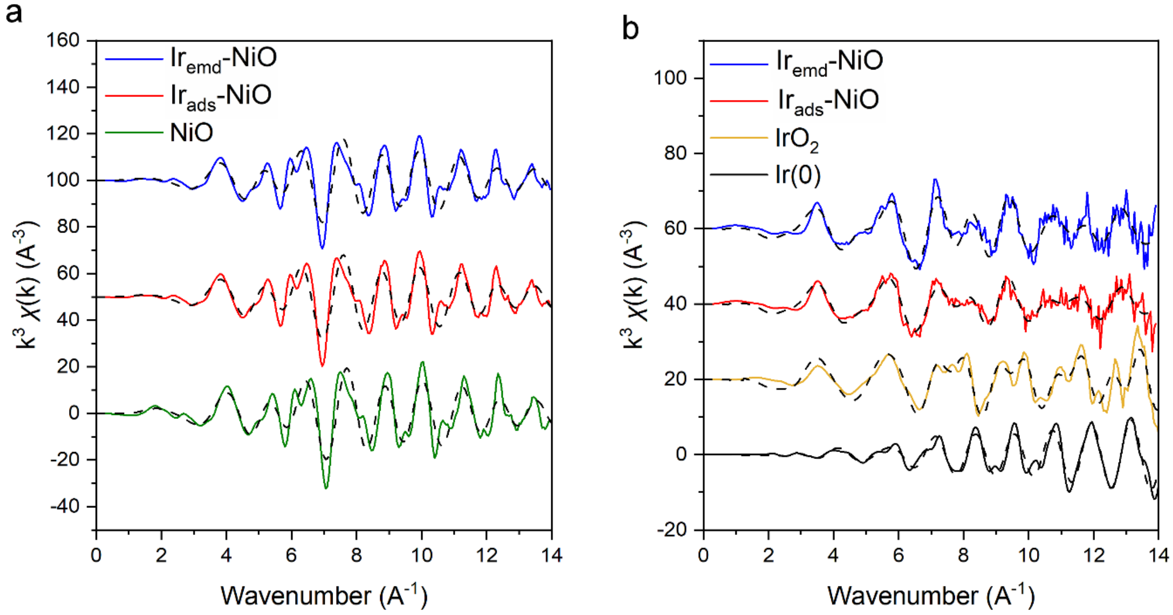

Figure S6. Experimental (solid line) and fitted (dashed line) of Ni *K*-edge (a) and Ir-L_3 edge (b) EXAFS spectra of Ir_{emb}-NiO and Ir_{ads}-NiO with other reference samples.

Table S2. EXAFS fitting parameters for Ni K-edge EXAFS region (S_0^2 fixed to 0.9).

Sample	Region	Shell	CN	R, Å	$\sigma^2(10^{-3})$, $Å^2$	E_0, eV	R-factor	Reduced Chi-square
**NiO**								
Ni K-edge	k = 3 – 14 $Å^{-1}$	Ni-O	6	2.07	5.3	8340.6	0.004	56
	R = 1.1 – 3 Å	Ni-Ni	12	2.95	5.6			
**Ir_{emb}-NiO**								
Ni K-edge	k = 3 – 14 $Å^{-1}$	Ni-O	6	2.07	5.5	8341.0	0.004	42
	R = 1.1 – 3 Å	Ni-Ni	12	2.95	6.0			
**Ir_{ads}-NiO**								
Ni K-edge	k = 3 – 14 $Å^{-1}$	Ni-O	6	2.07	5.8	8341.0	0.004	65
	R = 1.1 – 3 Å	Ni-Ni	12	2.95	6.0			

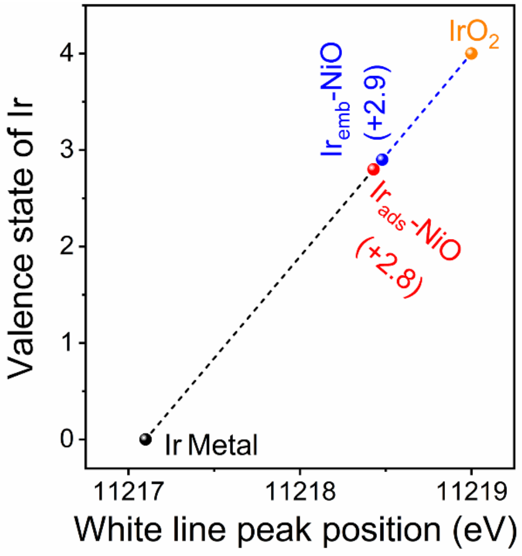

Figure S7. Ir oxidation state analysis by corresponding Ir-L_3-edge XANES white line peak position for Ir_{ads}-NiO and Ir_{emb}-NiO.

Table S3. EXAFS fitting parameters for Ir L_3-edge EXAFS region ($\mathbf{S_0}^2$ fixed to 0.9).

Sample	Region	Shell	CN	R, Å	σ^2(10^{-3}), $Å^2$	E_0, eV	R-factor	Reduced Chi-square
**Ir(0)**								
Ir L_3-edge	k = 3 – 13.5 $Å^{-1}$	Ir-Ir	12	2.71	4.4	11222.9	0.003	407
	R = 1.2 – 3 Å							
**IrO_2**								
Ir L_3-edge	k = 3 – 13.5 $Å^{-1}$	Ir-O	6	1.98	2.6	11225.8	0.079	92
	R = 1.2 – 4 Å	Ir-Ir	9	3.21	12.4			
		Ir-Ir	3	3.59	2.2			
**Ir_{ads}-NiO**								
Ir L_3-edge	k = 3 – 13.5 $Å^{-1}$	Ir-O	1.5	1.86	2.3	11222.4	0.044	38
	R = 1.2 – 3.5 Å	Ir-O	4.5	2.01	1.9			
		Ir-Ni	3.5	2.96	6.0			
		Ir-O	4	3.81	5.0			
**Ir_{emb}-NiO**								
Ir L_3-edge	k = 3 – 13.5 $Å^{-1}$	Ir-O	1.5	1.87	0.1	11221.0	0.053	42
	R = 1.2 – 3.5 Å	Ir-O	4.5	2.02	0.9			
		Ir-Ni	8	3.01	8.3			
		Ir-O	4	3.74	6.1			
**Ir_{emb}-NiO after stability test for 20 h**								
Ir L_3-edge	k = 3 – 13.5 $Å^{-1}$	Ir-O	1	1.85	2.2	11221.8	0.043	94
	R = 1.2 – 3.5 Å	Ir-O	5	1.99	1.6			
		Ir-Ni	8	3.01	8.1			
		Ir-O	4	3.81	4.6			
**$Ni_{0.98}Ir_{0.02}O$**								
Ir L_3-edge	k = 3 – 13.5 $Å^{-1}$	Ir-O	2	1.88	1.7	11221.3	0.045	26
	R = 1.2 – 3.5 Å	Ir-O	4	2.01	1.0			
		Ir-Ni	11	3.01	8.9			
		Ir-O	4	3.73	8.9			

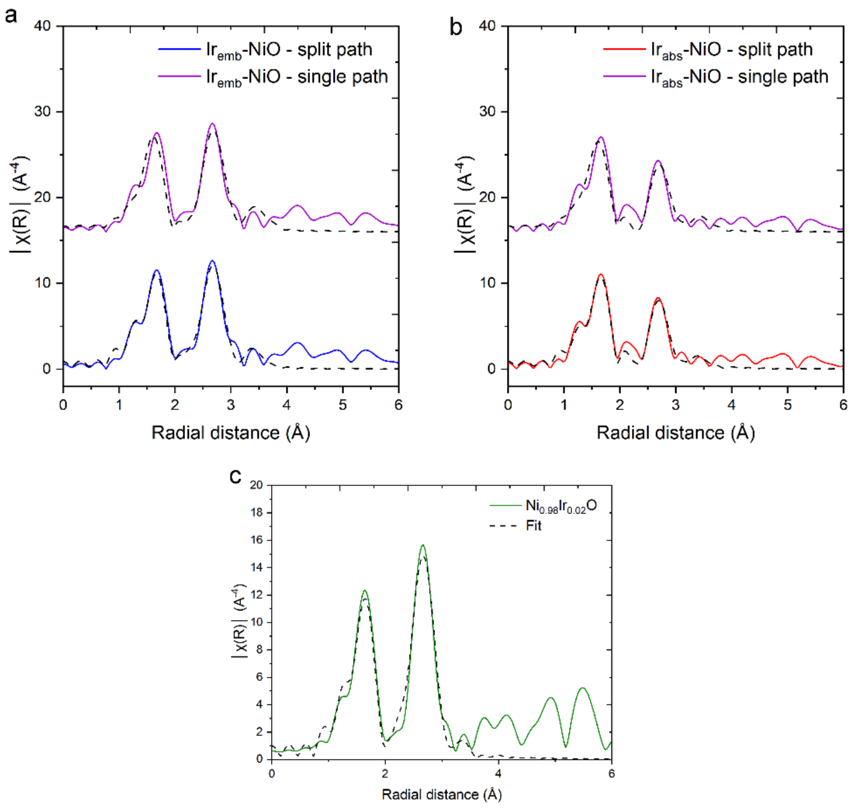

Figure S8. Experimental Ir-L_3 edge FT-EXAFS spectra of Ir_{emb}-NiO (a) and Ir_{ads}-NiO (b) samples. Experimental (solid lines) and fitted (dashed lines) fitted using a single path (purple lines) and a splitted path (blue and red lines) model for the first shell. (c) Experimental (solid line) and fitted (dashed line) Ir L_3-edge FT-EXAFS spectra of $Ni_{0.98}Ir_{0.02}O$.

Table S4. Comparison of EXAFS fitting parameters for Ir L_3-edge EXAFS region for Ir_{emb}-NiO and Ir_{ads}-NiO using split and single paths models for the first shell ($\mathbf{S_0}^2$ fixed to 0.9).

Sample	Region	Shell	CN	R, Å	$\sigma^2(10^{-3})$, Å^2	E_0, eV	R-factor	Reduced Chi-square
**Ir_{ads}-NiO (Split paths for first shell)**								
Ir L_3-edge	k = 3 – 13.5 Å^{-1}	Ir-O	1.5	1.86	2.3	11222.4	0.044	38
	R = 1.2 – 3.5 Å	Ir-O	4.5	2.01	1.9			
		Ir-Ni	3.5	2.96	6.0			
		Ir-O	4	3.81	5.0			
**Ir_{ads}-NiO (Single path for first shell)**								
Ir L_3-edge	k = 3 – 13.5 Å^{-1}	Ir-O	6	2.01	5.8	11224.3	0.064	40
	R = 1.2 – 3.5 Å	Ir-Ni	3.5	3.04	5.9			
		Ir-O	4	3.62	3.5			
**Ir_{emb}-NiO (Split paths for first shell)**								
Ir L_3-edge	k = 3 – 13.5 Å^{-1}	Ir-O	1.5	1.87	0.1	11221	0.053	42
	R = 1.2 – 3.5 Å	Ir-O	4.5	2.02	0.9			
		Ir-Ni	8	3.01	8.3			
		Ir-O	4	3.74	6.1			
**Ir_{emb}-NiO (Single path for first shell)**								
Ir L_3-edge	k = 3 – 13.5 Å^{-1}	Ir-O	6	2.01	5.2	11223.4	0.075	43
	R = 1.2 – 3.5 Å	Ir-Ni	8	3.02	8.4			
		Ir-O	4	3.86	0.7			

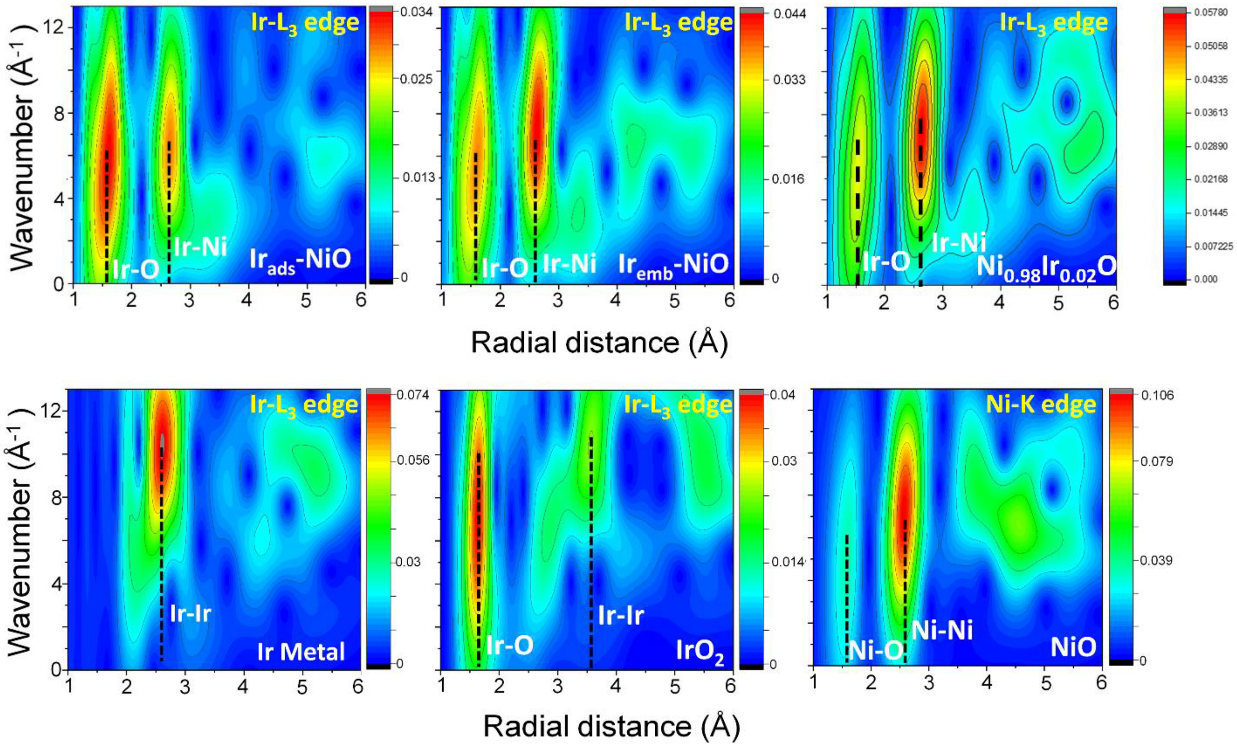

Figure S9. WT-EXAFS of Ir_{emb}-NiO, Ir_{ads}-NiO and $Ni_{0.98}Ir_{0.02}O$ along with Ir(0) and IrO_2 at Ir-L_3 edge compared with pristine NiO at Ni K-edge.

The WT-EXAFS analysis of Ir_{ads}-NiO, Ir_{emb}-NiO, and $Ni_{0.98}Ir_{0.02}O$ revealed a progressive increase in the intensity of the Ir-Ni second-shell peak. This trend is consistent with the rising Ir-Ni coordination number, from 3.5 in Ir_{ads}-NiO to 8 in Ir_{emb}-NiO and 11 in $Ni_{0.98}Ir_{0.02}O$, which corresponds to the increasing extent of Ir atom incorporation into the NiO matrix.

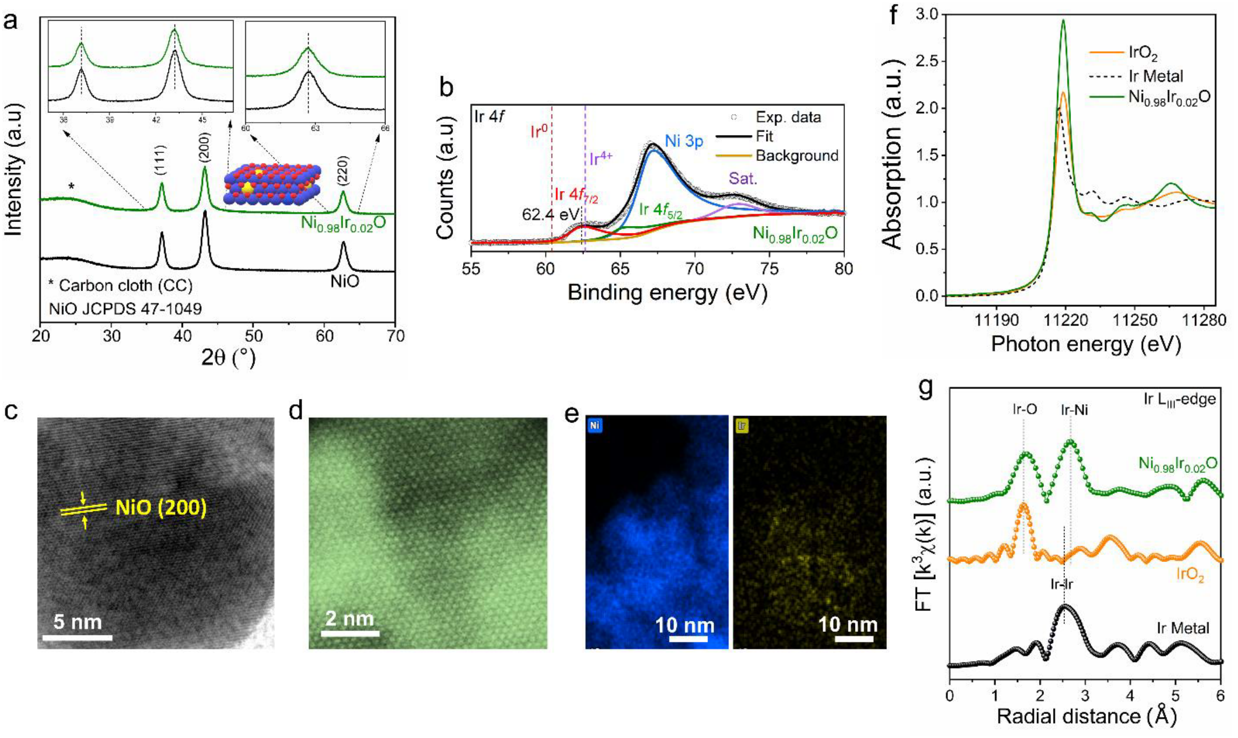

Figure S10. XRD pattern (a) and high-resolution Ir 4*f* XPS spectra (b) of $Ni_{0.98}Ir_{0.02}O$. Inset in (a) shows the enlarged view of the (111), (200) and (220) reflections. HAADF-STEM images at low-magnification (c), high-magnification (d) and corresponding elemental mapping (left: Ni and right: Ir) (e) of $Ni_{0.98}Ir_{0.02}O$. (f) Experimental Ir L_3-edge XANES spectra of $Ni_{0.98}Ir_{0.02}O$ with reference samples. (g) Experimental Ir L_3-edge FT-EXAFS spectra of $Ni_{0.98}Ir_{0.02}O$ with reference samples.

The overlapping XRD pattern of $Ni_{0.98}Ir_{0.02}O$ with NiO suggests the presence of ~6 wt% Ir atoms into the NiO lattice (Figure S10a and Table S1). No significant peak shifts were detected following Ir substitution into the NiO lattice, likely due to the low Ir content (only 2%) within the NiO matrix and the comparable ionic radii of Ir^{3+} and Ni^{2+} in octahedral geometries (inset, Figure S10a). The high-resolution Ir $4f_{7/2}$ XPS spectra of $Ni_{0.98}Ir_{0.02}O$ revealed that the oxidation state of Ir was situated between those of 0 and +4, whereas the significantly low intensity of the Ir $4f_{7/2}$ peak compared to Ir_{ads}-NiO and Ir_{emb}-NiO suggested that most of the Ir atoms are into the lattice of NiO and very small fraction of Ir were detected on the surface by surface-sensitive XPS analysis (Figure S10b). Atomic-scale AC-HAADF-STEM images of $Ni_{0.98}Ir_{0.02}O$ displayed a well-ordered NiO matrix, though isolated Ir atoms on the surface were difficult to detect due to their minimal surface presence. Most of the Ir atoms were incorporated into the NiO lattice, as supported by XRD and

XPS analyses (Figure S10c,d). EDS mapping (Figure S10e) further confirmed the homogeneous distribution of Ir within the NiO lattice. Consistent with XRD and XPS findings, Ir L_3-edge XAS analysis (XANES and FT-EXAFS) of $Ni_{0.98}Ir_{0.02}O$ revealed a higher oxidation state for the substituted Ir atoms, with distinct Ir-O and Ir-Ni coordination patterns typical of conventional substitution in the NiO crystal, and confirmed the absence of metallic nanoparticles (Figure S10f,g).

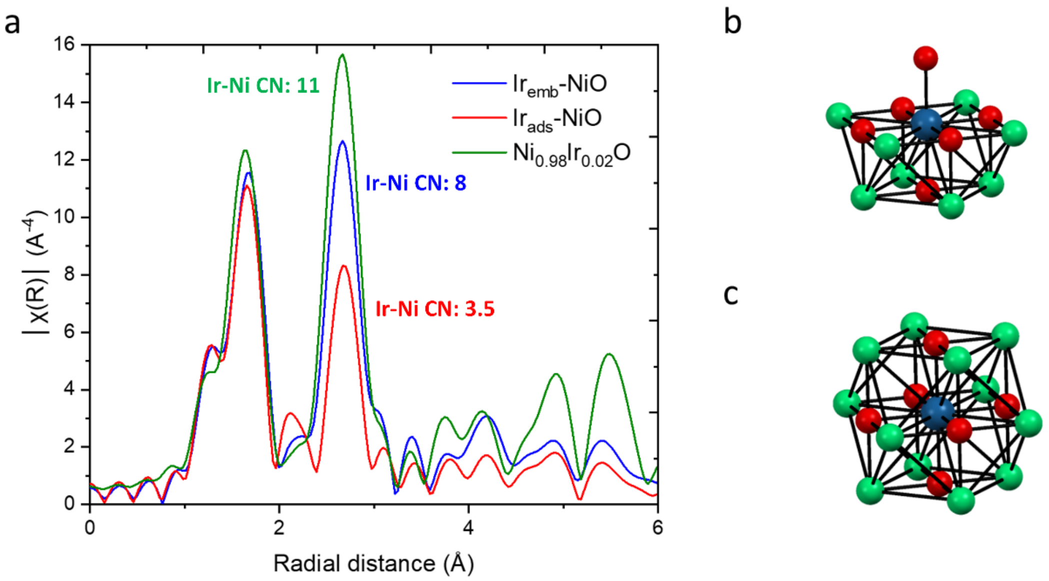

Figure S11. Comparison of Ir-L_3 edge FT-EXAFS spectra of Ir_{ads}-NiO, Ir_{emb}-NiO and $Ni_{0.98}Ir_{0.02}O$ (a). Expected coordination environment for superficial and internal Ir atoms (b and c, respectively) in the NiO structure. Color code: Ir atoms (blue), Ni atoms (green) O atoms (red).

Figure S11a demonstrates the effective modulation of the Ir-Ni second coordination shell, with coordination numbers increasing from 3.5 in Ir_{ads}-NiO to 8 and 11 in Ir_{emb}-NiO and $Ni_{0.98}Ir_{0.02}O$, respectively. This progressive increase in Ir-Ni coordination reflects the varying degrees of Ir atom anchoring, ranging from surface adsorption to partial encapsulation and eventual lattice incorporation (Figure S11b,c).

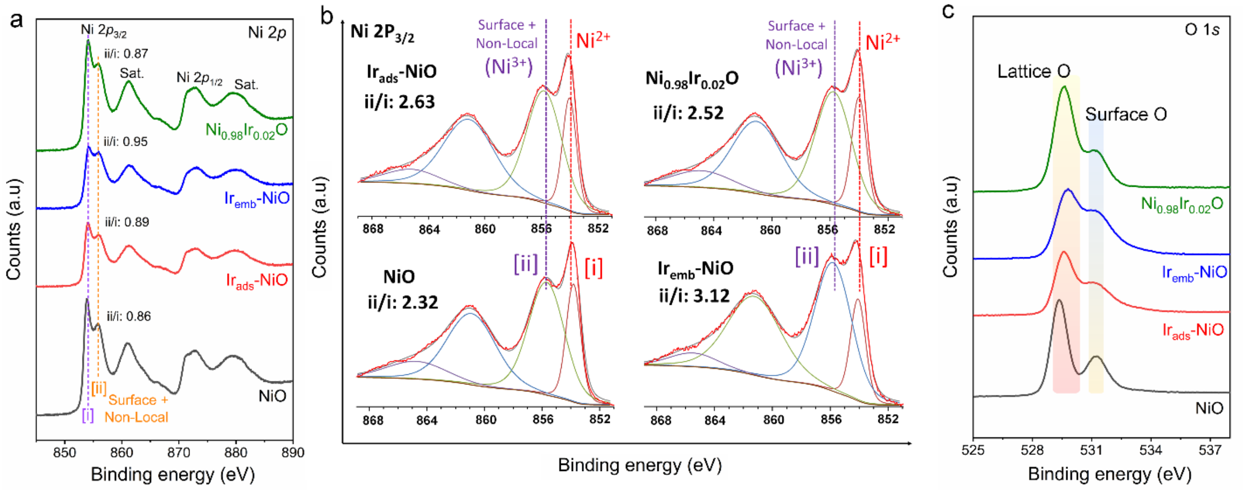

Figure S12. High-resolution Ni 2*p* XPS spectra (a), fitted deconvoluted high-resolution Ni $2p_{3/2}$ XPS spectra (b) and O 1*s* XPS spectra (c) of Ir_{ads}-NiO and Ir_{emb}-NiO with reference samples.

The dominant peak observed in the Ni $2p_{3/2}$ XPS spectra presents multiple sub-peaks labeled as 'i' (Ni^{2+}) and 'ii' (Ni^{3+}) at ~854 and ~856 eV, respectively (Figure S12a).[S1] The increased ratio of integrated areas for ii/i in Ir_{emb}-NiO, compared to pristine NiO and Ir_{ads}-NiO, indicates a higher valence state of surface Ni atoms. This elevation is attributed to the enhanced electronic coupling between the partially encapsulated Ir and surface Ni atoms, driven by the increased Ir-Ni coordination number (Figure S12b). For $Ni_{0.98}Ir_{0.02}O$, despite exhibiting the highest Ir-Ni coordination number, the ii/i ratio is only slightly higher than that of pristine NiO and significantly lower than that of Ir_{emb}-NiO. This suggests only a modest increase in the valence state of surface Ni atoms, likely due to the minimal surface presence of Ir atoms, as indicated by Ir 4f XPS. Consequently, the electronic coupling between Ir and surface Ni atoms is also less pronounced.

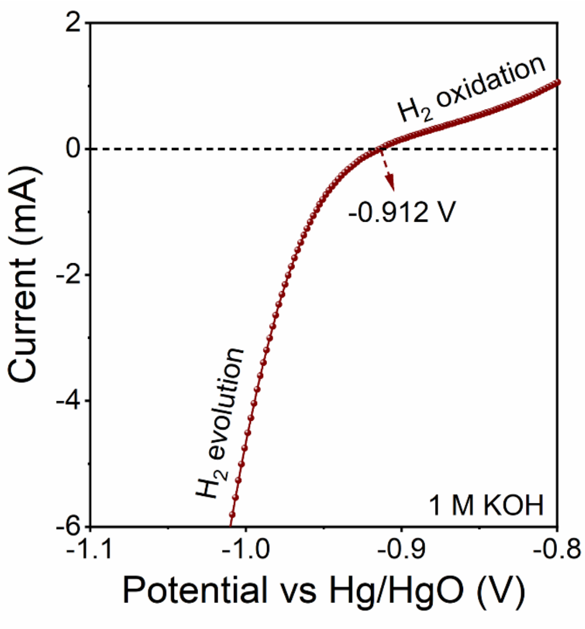

Figure S13. Hg/HgO reference electrode calibration in H_2 saturated 1 M KOH solution.

The potential of the Hg/HgO reference electrode was calibrated in H_2 saturated 1 M KOH solution using Pt mesh as both working and counter electrode. The LSV curve were recorded at a scan rate of 5 mV/s. The potential achieved at the zero current is the thermodynamic potential for H_2 evolution/oxidation. Here, the zero current was achieved at -0.912 V vs Hg/HgO, and therefore $E_{RHE} = E_{Hg/HgO} + 0.912$ V.

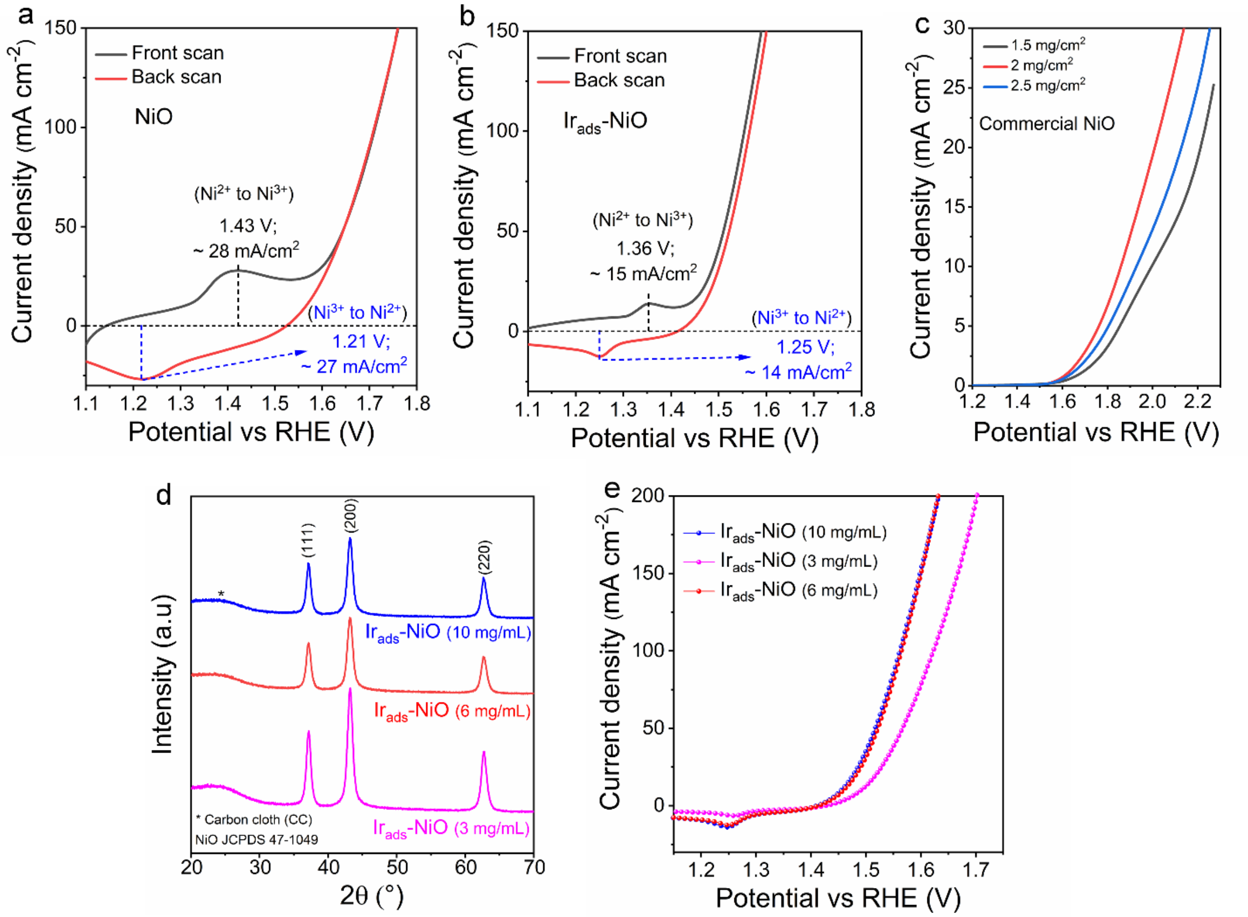

Figure S14. CV curves for NiO (a) and Ir_{ads}-NiO (b) in 1 M KOH. (c) LSV polarization curves of commercial NiO with different catalysts loadings in 1 M KOH. XRD pattern (d) and alkaline OER LSV polarization curves (e) of Ir_{ads}-NiO obtained using different concentrations of Ir-EtOH solution (3, 6, and 10 mg/mL).

The shift in the Ni^{2+}/Ni^{3+} peak potential to lower values for Ir_{ads}-NiO compared to pristine NiO suggests an enhanced metal-support interaction between surface Ir atoms and the NiO support (Figure S14a,b). Additionally, the reduced Ni^{2+}/Ni^{3+} peak area further corroborates the diminished presence of surface Ni^{2+} species following the stabilization of Ir single atoms, aligning with the Ni $2p_{3/2}$ XPS analysis. Figure S14c presents the LSV polarization curves in 1 M KOH for commercial NiO with varying catalyst loadings, demonstrating that ~2 mg/cm² loading on carbon cloth provides an optimal balance between the number of active sites and mass diffusion. Figure S14d illustrates the XRD pattern of Ir_{ads}-NiO synthesized employing various concentrations of Ir-EtOH solution (3, 6, and 10 mg/mL). The diffraction peaks align closely with those of NiO, indicating

the atomic dispersion of Ir sites and the absence of Ir-based nanoparticles. Additionally, the LSV polarization curves demonstrate that Ir_{ads}-NiO (6 mg/mL), synthesized with a 6 mg/mL Ir-EtOH solution, exhibited optimized acidic OER activity (Figure S14e).

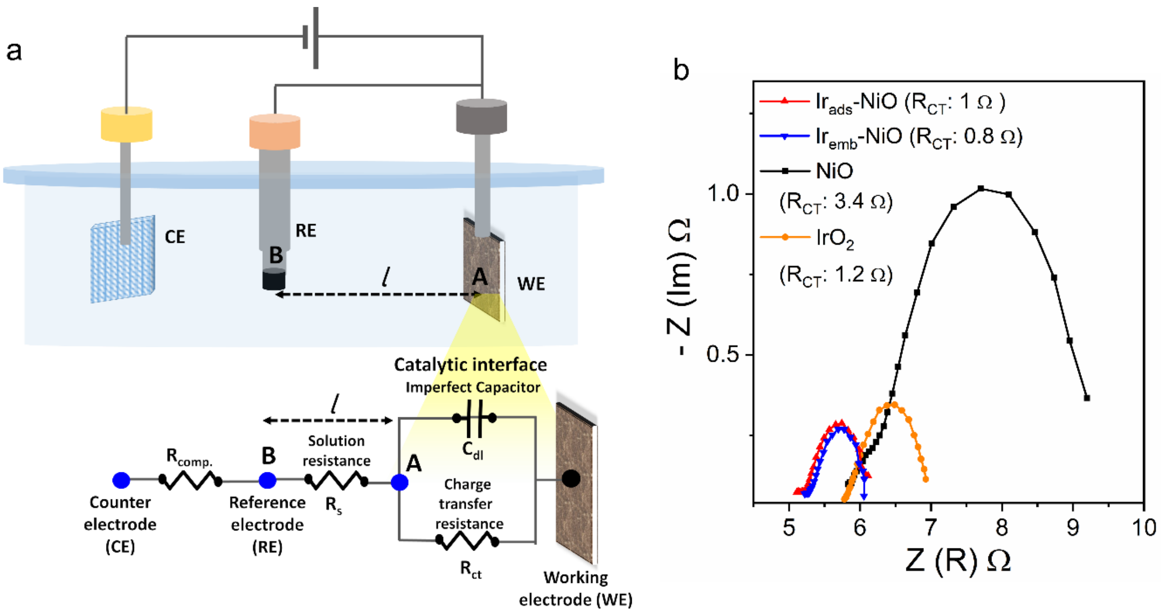

Figure S15. (a) Three-electrode setup with the corresponding equivalent circuit diagram. (b) Nyquist plot for OER at 494 mV overpotential in 1 M KOH.

Figure S15a illustrates the typical three-electrode setup used for electrochemical measurements, along with the corresponding equivalent circuit diagram. The resistance between the working and reference electrodes (denoted as points A and B) is identified as the solution resistance (R_s). The R_s value is influenced not only by the concentration of the solution but also by the distance between the tip of the reference electrode and the center of the working electrode. Even slight variations in the distance between points A and B can impact the R_s value. As shown in Figure S15b, all the catalysts exhibit slight variations in R_s, likely due to the manual immersion of the electrodes, leading to differences in the spacing between the reference and working electrodes. Nevertheless, all the LSV curves presented have been *iR*-corrected based on the corresponding R_s values to ensure an effective comparison.

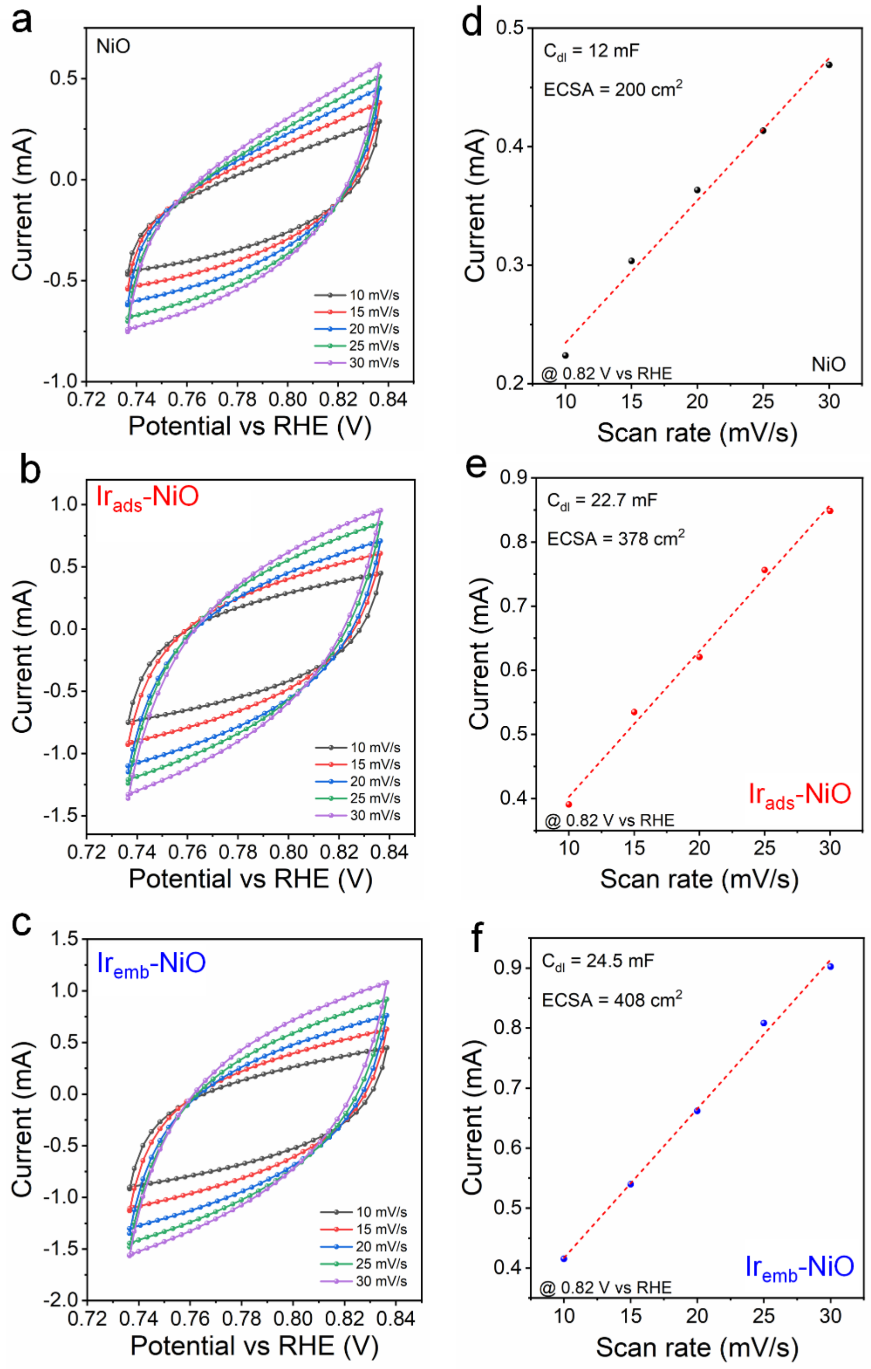

Figure S16. CV plots of NiO (a), Ir_{ads}-NiO (b), and Ir_{emb}-NiO (c), at different scan rates. Current density (recorded at a fixed potential) as a function of scan rate for NiO (d), Ir_{ads}-NiO (e), and Ir_{emb}-NiO (f).

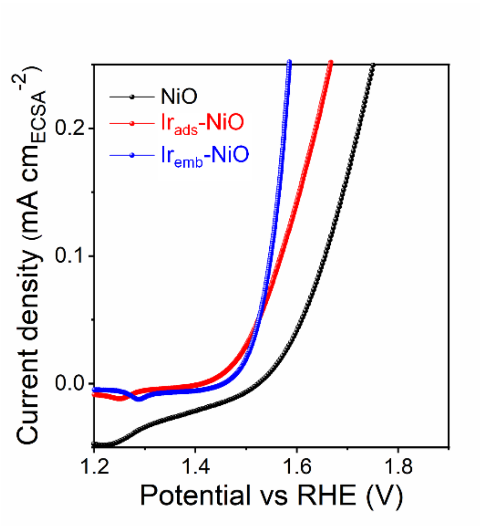

Figure S17. ECSA normalized alkaline OER LSV polarization curve in 1 M KOH.

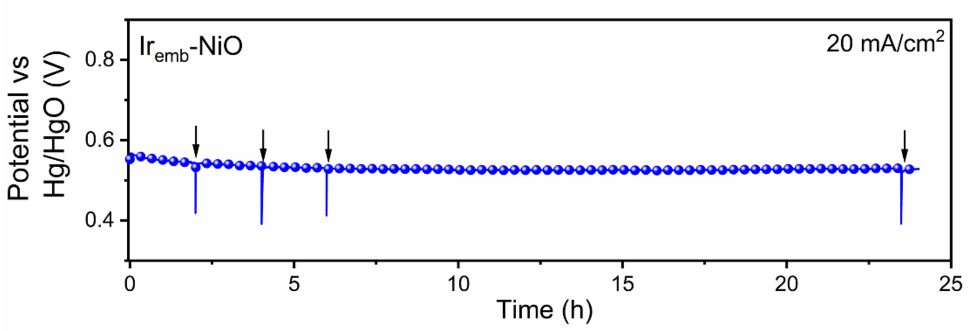

Figure S18. Chronopotentiometric test of Ir_{emb}-NiO at 20 mA/cm^2 for analyzing the dissolved ions in the electrolyte by ICP-OES. (The black arrow points to the time of electrolyte collection).

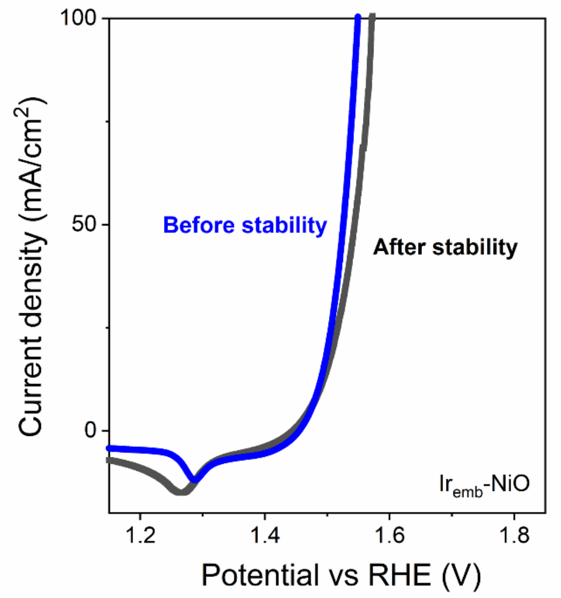

Figure S19. Alkaline OER LSV polarization curve before and after the stability test of Ir_{emb}-NiO.

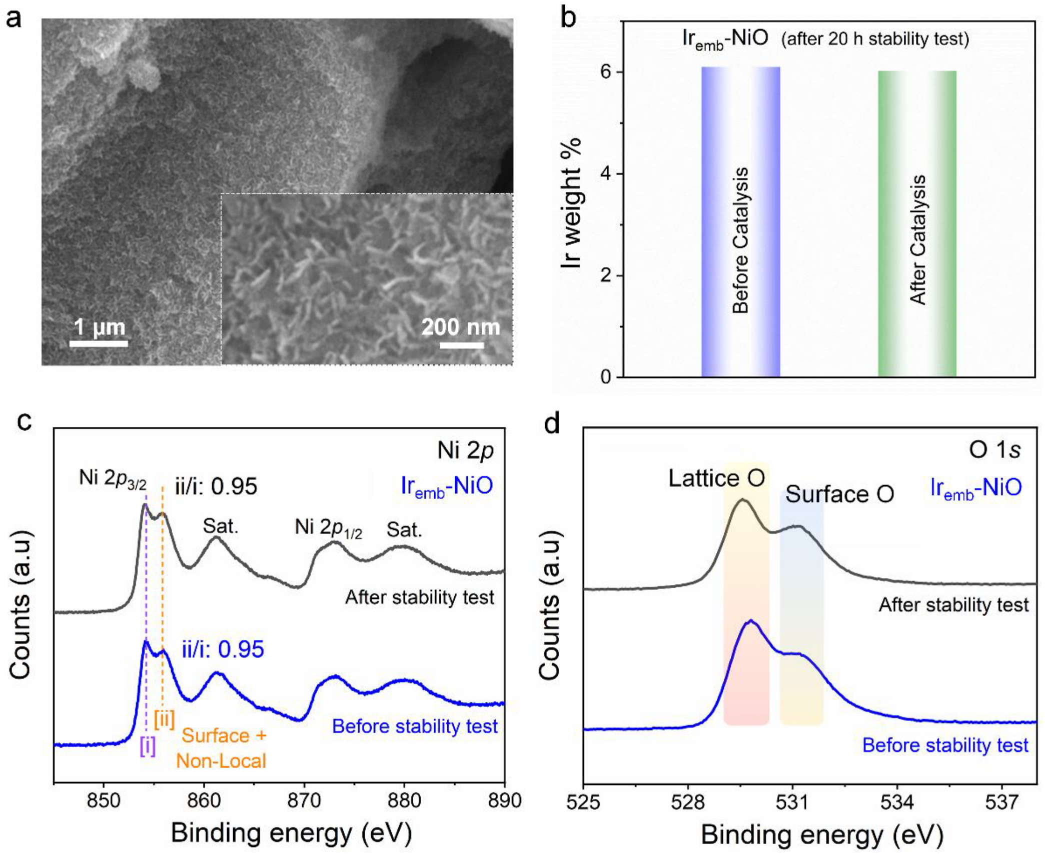

Figure S20. FESEM image (a), and Ir weight % from ICP-OES analysis (b) of Ir$_{emb}$-NiO after 20 h of stability test. High-resolution Ni 2p XPS spectra (c), and high-resolution O 1s XPS spectra (d) of Ir$_{emb}$-NiO before and after the 20 h of stability test.

The post stability FESEM image of Ir$_{emb}$-NiO showed negligible morphological modification, and the ICP-OES analysis revealed a similar Ir loading compared to the fresh sample (Figure S20a,b). The Ni 2*p* XPS spectra revealed negligible changes after the stability test, while the O1*s* XPS spectra showed a slight increase in the surface O species after the OER stability test (Figure S20c,d).

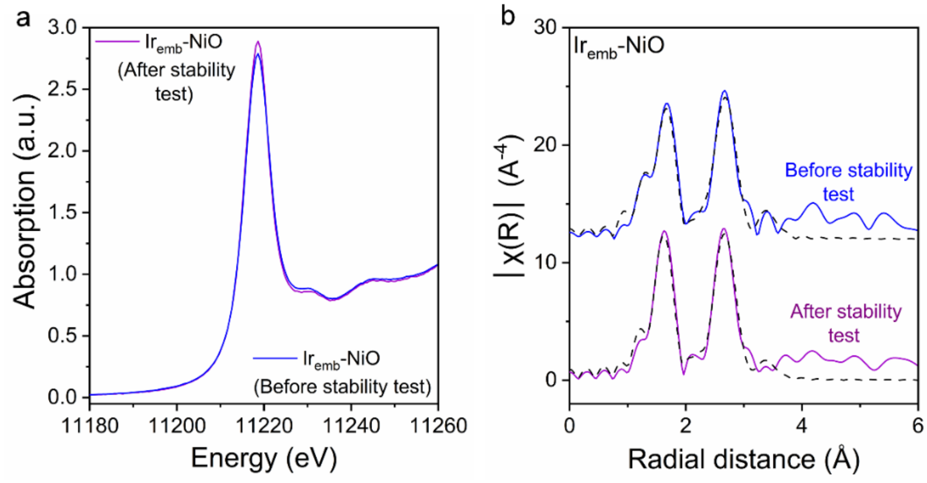

Figure S21. (a) Experimental Ir L_3-edge XANES spectra of Ir_{emb}-NiO before and after the stability test for 20 h. (b) Experimental (solid lines) and fitted (dashed lines) Ir L_3-edge FT-EXAFS spectra of Ir_{emb}-NiO before and after the stability test for 20 h.

a

Electrolyte	Ni (ppb)	Fe (ppb)	Ir (ppb)
1 M comm. KOH solution (fresh)	0	75	0

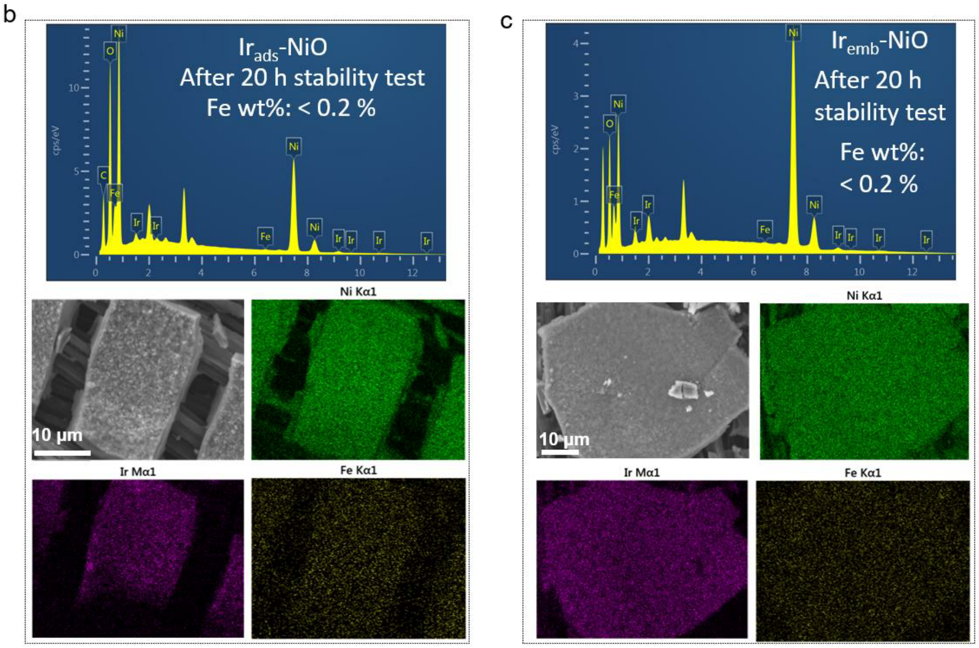

Figure S22. (a) ICP-OES analysis of the fresh electrolyte (1 M KOH). The EDS pattern and the corresponding mapping of Ni, Ir and Fe for Ir_{ads}-NiO (b) and Ir_{emb}-NiO (c) after the 20 h of the stability test.

Table S5. ICP-OES analysis of the electrolyte before and after the long-tern stability test for 110 h.

Electrolyte	Ni (ppb)	Ir (ppb)
Fresh alkaline solution (comm. KOH)	0	0
Electrolyte after 110 h of OER stability test	166	74
Initial metal loading (Ir_{emb}-NiO)	26800	1760
Relative metal dissolution (%) after long-tern durability test	~0.62 %	~4.2 %

After the long-term stability test, only approximately 4% of the active Ir sites were lost, while the NiO substrate showed minimal degradation of around 0.6%. This highlights the robustness of the synthesized catalysts, attributed to the increased Ir-Ni second-shell coordination, which effectively prevents the leaching of active Ir sites from the NiO surface.

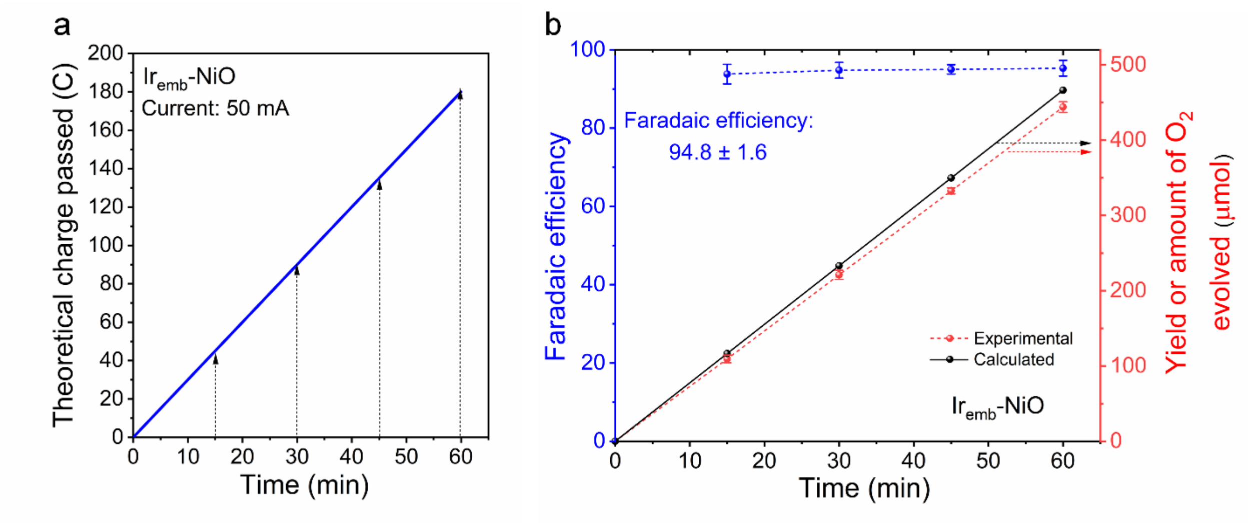

Figure S23. (a) Theoretical charge passed over time at a constant current of 50 mA for Ir_{emb}-NiO. (b) Faradaic efficiency of Ir_{emb}-NiO showing the theoretically calculated and experimentally measured O_2 gas with time.

The Faradaic efficiency was determined by measuring the actual amount of oxygen gas produced by Ir_{emb}-NiO during the OER at a constant current of 50 mA, using the water displacement method. The calculated Faradaic efficiency of Ir_{emb}-NiO for the alkaline OER is 94.8 ± 1.6 % (Figure S23). Figure S23b illustrates the yield or the actual amount of O_2 evolved over time at a constant current of 50 mA.

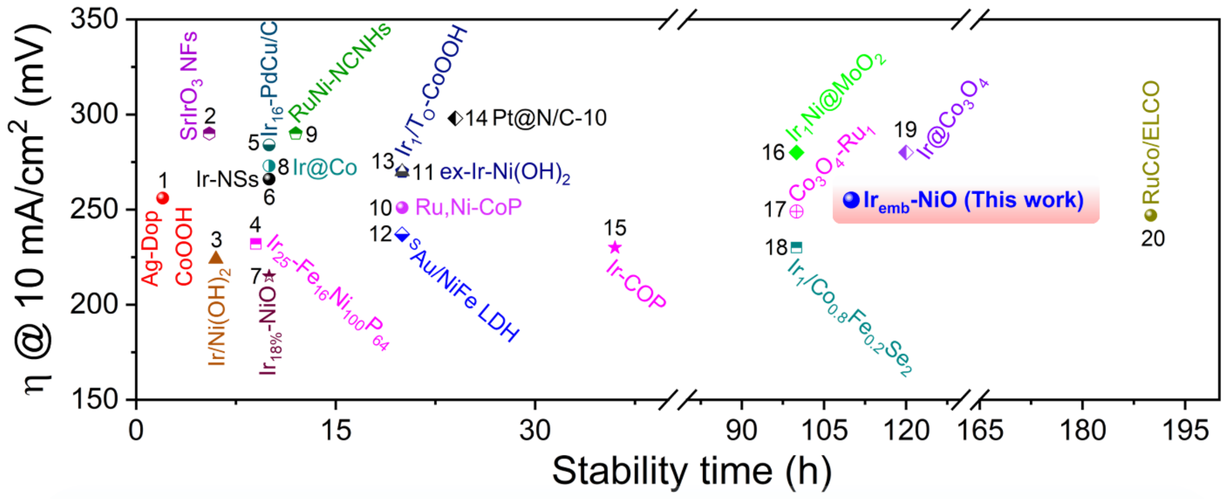

(1) *ACS Catal.* **2020**, *10*, 562.
(2) *ACS Appl. Energy Mater.* **2022**, *5*, 6146.
(3) *Adv. Mater.* **2020**, *32*, 2000872.
(4) *Small* **2023**, *19*, 2207253.
(5) *Nano Lett.* **2021**, *21*, 5774.
(6) *Nat. Sci. Rev.* **2020**, *7*, 1340.
(7) *J. Am. Chem. Soc.* **2020**, *142*, 7425.
(8) *J. Mater. Chem. A* **2019**, *7*, 8376.
(9) *Adv.Sci.* **2020**, *7*, 1901833.
(10) *Appl. Catal. B* **2021**, *298*, 120488.
(11) *ACS Catal.* **2021**, 11, 5386.
(12) *J. Am. Chem. Soc.* **2018**, *140*, 3876.
(13) *Nat. Commun.* **2022**, *13*, 2473.
(14) *Chinese J. Catal.* **2020**, *41*, 839.
(15) *Adv. Funct. Mater.* **2023**, *33*, 2211192.
(16) *Adv. Mater.* **2023**, 2305437.
(17) *Nano Today* **2020**, *34*, 100955 .
(18) *Nat. Commun.* **2020**, *11*, 1215.
(19) *Adv. Funct. Mater.* **2022**, *32*, 2111989.
(20) *Angew. Chem. Int. Ed.* **2022**, 61, *e2022059*.

Figure S24. Comparison of the alkaline OER activity and stability of Ir_{emb}-NiO with other recently reported catalysts.

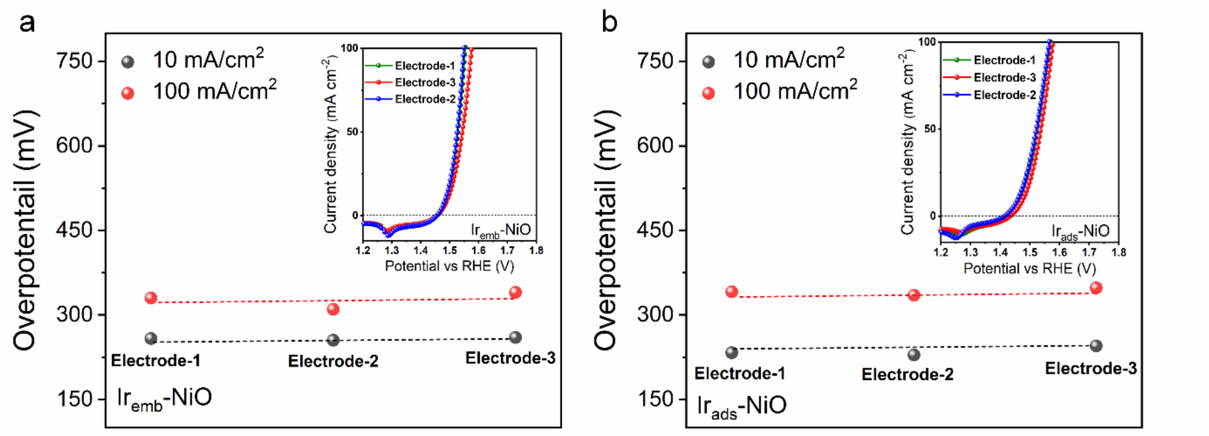

Figure S25. OER LSV polarization curves of three different electrodes for Ir_{emb}-NiO (a) and Ir_{ads}-NiO (b) in alkaline media.

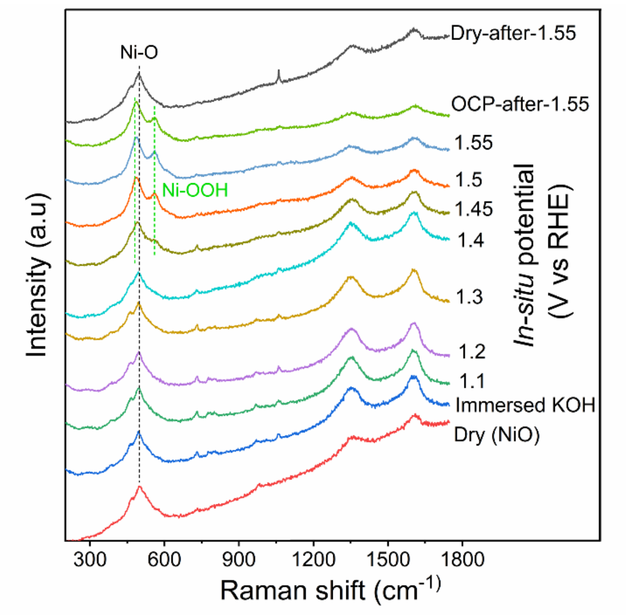

Figure S26. *In-situ* Raman spectroscopy measurements for NiO recorded during alkaline OER from 1.1 to 1.55 V vs RHE.

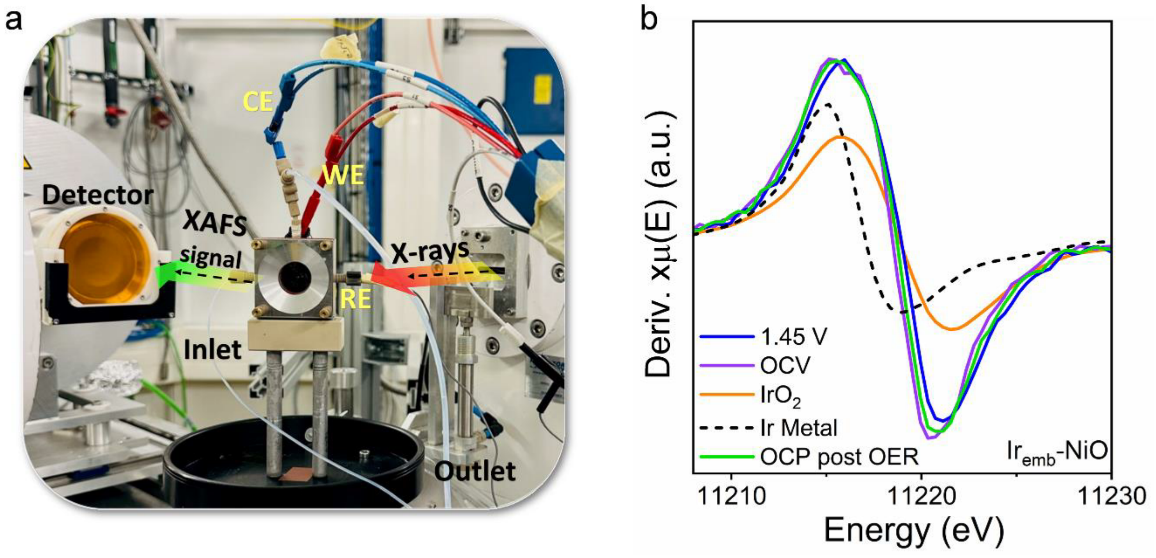

Figure S27. (a) Setup of the operando XAS measurements. The red arrow indicates the incident X-ray, while the green arrow indicates the fluorescence X-ray. (b) Derivative of the experimental Ir L_3-edge XANES spectra of Ir_{emb}-NiO at OCV, 1.45 V and OCV after the catalytic process in alkaline solution with Ir(0) and IrO_2 reference samples.

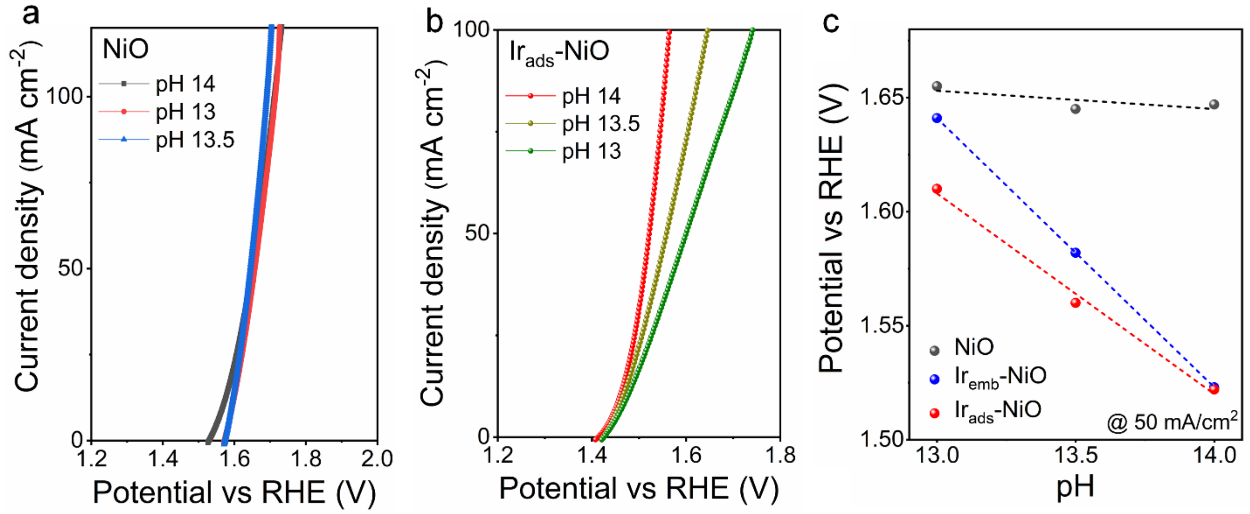

Figure S28. pH-dependent LSV polarization curves of NiO (a), and Ir_{ads}-NiO (b) recorded in KOH. (c) pH dependence of the OER potential on the RHE scale at 50 mA/cm^2 for Ir_{emb}-NiO, Ir_{ads}-NiO, and NiO.

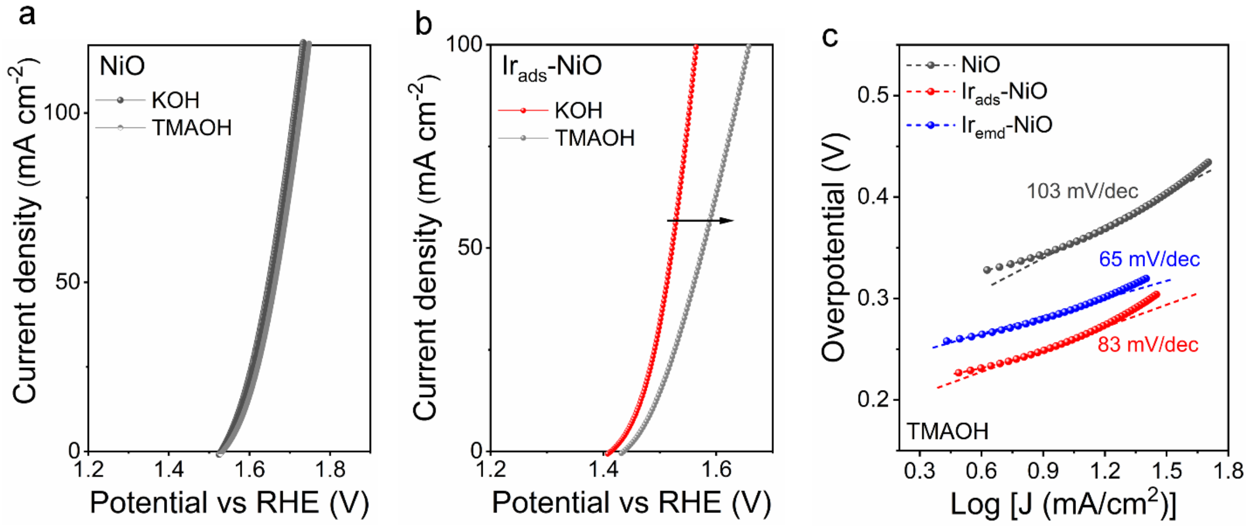

Figure S29. LSV polarization curves of NiO (a), and Ir_{ads}-NiO (b) recorded in 1 M KOH and TMAOH. (c) Tafel plots of Ir_{emb}-NiO, Ir_{ads}-NiO, and NiO in TMAOH.

References:

S1. Cheng, M.; Fan, H.; Song, Y.; Cui, Y.; Wang, R. Interconnected hierarchical $NiCo_2O4$ microspheres as high-performance electrode materials for supercapacitors. *Dalton Trans*. **2017**, *46*, 9201– 9209.
